# Multiplexed base editing through Cas12a variant-mediated cytosine and adenine base editors

**DOI:** 10.1038/s42003-022-04152-8

**Published:** 2022-11-02

**Authors:** Fangbing Chen, Meng Lian, Bingxiu Ma, Shixue Gou, Xian Luo, Kaiming Yang, Hui Shi, Jingke Xie, Weika Ge, Zhen Ouyang, Chengdan Lai, Nan Li, Quanjun Zhang, Qin Jin, Yanhui Liang, Tao Chen, Jiaowei Wang, Xiaozhu Zhao, Lei Li, Manya Yu, Yinghua Ye, Kepin Wang, Han Wu, Liangxue Lai

**Affiliations:** 1 CAS Key Laboratory of Regenerative Biology, Guangdong Provincial Key Laboratory of Stem Cell and Regenerative Medicine, Guangzhou Institutes of Biomedicine and Health, Chinese Academy of Sciences, Guangzhou, 510530 China; 2 Institute of Laboratory Animal Sciences, Chinese Academy of Medical Sciences & Peking Union Medical College, Beijing, 100730 China; 3 Research Unit of Generation of Large Animal Disease Models, Chinese Academy of Medical Sciences (2019RU015), Guangzhou, 510530 China; 4 Department of Gynecology and Obstetrics, Key Laboratory for Major Obstetric Diseases of Guangdong Province, Key Laboratory of Reproduction and Genetics of Guangdong Higher Education Institutes, The Third Affiliated Hospital of Guangzhou Medical University, Guangzhou, 510150 China; 5 Sanya Institute of Swine Resource, Hainan Provincial Research Center of Laboratory Animals, Sanya, 572000 China; 6 Guangdong Provincial Key Laboratory of Large Animal models for Biomedicine, Wuyi University, Jiangmen, 529020 China; 7 Joint School of Life Sciences, Guangzhou Institutes of Biomedicine and Health, Chinese Academy of Sciences, Guangzhou Medical University, Guangzhou, 511436 China; 8 Bioland Laboratory (Guangzhou Regenerative Medicine and Health Guangdong Laboratory), Guangzhou, 510005 China; 9 University of Chinese Academy of Sciences, Beijing, 100049 China

**Keywords:** Genetic engineering, CRISPR-Cas9 genome editing

## Abstract

Cas12a can process multiple sgRNAs from a single transcript of CRISPR array, conferring advantages in multiplexed base editing when incorporated into base editor systems, which is extremely helpful given that phenotypes commonly involve multiple genes or single-nucleotide variants. However, multiplexed base editing through Cas12a-derived base editors has been barely reported, mainly due to the compromised efficiencies and restricted protospacer-adjacent motif (PAM) of TTTV for wild-type Cas12a. Here, we develop Cas12a-mediated cytosine base editor (CBE) and adenine base editor (ABE) systems with elevated efficiencies and expanded targeting scope, by combining highly active deaminases with *Lachnospiraceae bacterium* Cas12a (LbCas12a) variants. We confirm that these CBEs and ABEs can perform efficient C-to-T and A-to-G conversions, respectively, on targets with PAMs of NTTN, TYCN, and TRTN. Notably, multiplexed base editing can be conducted using the developed CBEs and ABEs in somatic cells and embryos. These Cas12a variant-mediated base editors will serve as versatile tools for multiplexed point mutation, which is notably important in genetic improvement, disease modeling, and gene therapy.

## Abstract (web summary)

Cas12a-mediated cytosine (CBE) and adenine base editor (ABE) systems with elevated efficiencies and expanded target scopes are developed, by combining highly active deaminases with Cas12a variants, enCas12a, RR and RVR, recognizing non-TTTV PAMs.

## Introduction

Base editors, including cytosine base editors (CBEs)^1,2^ and adenine base editors (ABEs)^3^, are newly developed CRISPR/Cas-based genome modified tools that combine catalytically impaired Cas nucleases with different kinds of deaminases. CBEs and ABEs can precisely and efficiently convert C-to-T and A-to-G, respectively, at single-base resolution^4^. Since their development, base editors have been widely adopted in various organisms to induce point mutations to mimic disease-causative mutations and agronomically important variations^5,6^. Given that numerous biological traits and diseases involve multiple single-nucleotide variants (SNVs) at different positions or multiple genes^7,8^, simultaneous base editing of multiple loci is urgently needed for disease modeling or crop improvement.

Cas12a, a member of the type V CRISPR family formerly called Cpf1^9^, can process multiple sgRNAs (also CRISPR RNAs (crRNAs)) from a single array transcript through its intrinsic RNase activity, thus conferring the flexible capacity of multi-gene targeting^10–15^. In virtue of the recognition of thymidine (T)-rich protospacer-adjacent motif (PAM) sequences, the catalytically inactivated dead Cas12a (dCas12a) has been adopted for the development of base editors to expand the targeting scope^16^. However, studies rarely reported Cas12a-mediated multiplexed base editing systems. The use of Cas12a for multiple base editing is impeded by poor efficiencies and strict PAM requirement, as the common *Lachnospiraceae bacterium* Cas12a (LbCas12a) and *Acidaminococcus sp*. Cas12a (AsCas12a) recognize canonical TTTV PAM sequences (where V is A, C, or G)^9^. Cas12a variants developed recently by structure-guided protein engineering, including RR, RVR^17^, enAsCas12a^18^, and impLbCas12a^19^, enable the recognition of alternative non-canonical PAMs, whereas extremely low base editing efficiencies hinder their practical applications, especially for multiple loci editing. Thus, versatile Cas12a-mediated base editor systems that can execute efficient editing of targets with non-canonical PAMs in addition to TTTV are urgently needed, which will be favorable for multiplexed base editing.

Although the compromised efficiencies of Cas12a-mediated base editors are likely in part due to the incapability to generate Cas12a nickase^1–3,16^, we and several other groups have demonstrated recently that the efficiencies of dCas12a-mediated CBEs and ABEs can be improved substantially with highly active deaminases on targets with TTTV PAM sequences^20–23^. In this study, we sought to develop LbCas12a (hereafter abbreviated as Cas12a) variant-mediated robust CBEs and ABEs with expanded target scope, which are capable of multiplexed base editing efficiently. By fusing highly active deaminases with engineered dCas12a variants, including RR, RVR, and enCas12a, we confirmed that these Cas12a variant-mediated CBEs and ABEs can act robustly at endogenous sites with canonical TTTV PAM and non-canonical PAMs, such as VTTV, TYCN, and TRTV (where Y is C or T, and R is A or G). Importantly, these base editors can performed multiplexed base editing in HEK293T cells and porcine embryos. With large improvements in efficiencies and targeting ranges, these newly developed Cas12a variant-derived CBEs and ABEs will serve as potent base editing tools and pave the way for multiplexed base editing to introduce or correct point mutations at several loci simultaneously, which is valuable in basic research, agriculture, and medicine.

## Results

### Robust C-to-T conversion by Cas12a variant-mediated CBEs for non-canonical PAMs

We initially attempted to screen for optimal cytosine deaminases that confer powerful C-to-T editing for Cas12a-mediated CBEs. Seven dCas12a-CBEs fused with individual cytosine deaminases^1,2,16,20–22,24–26^ were compared in HEK293T cells and porcine embryos at sites with TTTV PAM sequences. The dCas12a-A3A harboring human APOBEC3A (hA3A) exhibited superior efficiencies at most sites and different sequence contexts compared with other dCas12a-CBEs (Supplementary Fig. 1). Importantly, the introduction of the hA3A-Y130F or hA3A-Y132D variant^24^ to dCas12a-A3A could improve the accuracy of base editing by narrowing the extremely wide activity windows, from 10 nt (positions 6–15) to 6 nt (positions 7–12), and alleviate the potential toxic effect on embryo development due to the exceedingly high deaminase activity of hA3A while maintaining considerable C-to-T editing efficiencies (Supplementary Figs. 2, 3a). The introduction of the hA3A-N57G variant^27^ to dCas12a-A3A, on the other hand, conferred TCR or TCCR preference (Supplementary Fig. 3b), which can attain excellent accuracy when several Cs are located in the activity window. Therefore, the hA3A-Y130F and hA3A-N57G were selected for the construction of robust Cas12a-CBEs with expanded PAM compatibility.

Several AsCas2a variants have been reported to recognize alternative non-canonical PAMs^17,18^. To relieve the intrinsic limit of LbCas12a for TTTV PAM sequences to develop versatile Cas12a-mediated base editors with expanded target scope, we engineered LbCas12a by introducing certain amino acid mutations, based on its homology to AsCas12a, resulting in the generation of three LbCas12a variants, including RR (G532R/K595R), RVR (G532R/K538V/Y542R), and enCas12a (D156R/G532R/K538R) (Supplementary Fig. 4).

**Fig. 1:**
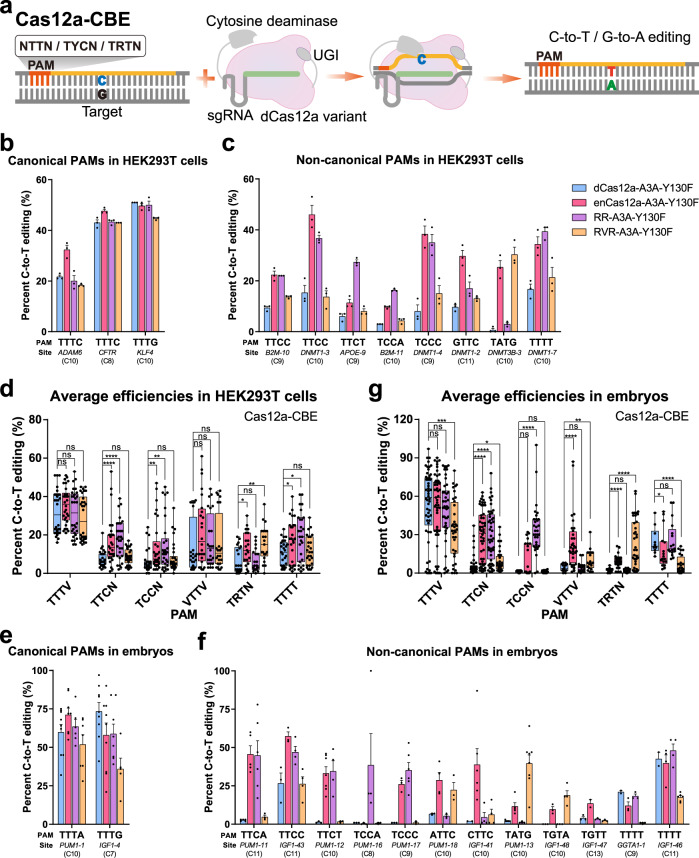
Efficient C-to-T conversion by Cas12a variant-mediated CBEs for non-canonical PAMs. **a** Schematic of C-to-T editing on targets with non-canonical PAMs by Cas12a variant-mediated CBEs. PAM protospacer-adjacent motif; UGI uracil DNA glycosylase inhibitor. Efficiencies of Cas12a variant-mediated CBEs, including dCas12a-, enCas12a-, RR-, and RVR-A3A-Y130F, for canonical TTTV PAM (**b**) and non-canonical PAMs (**c**) in HKE293T cells (*n* = 3). **d** Average efficiencies of Cas12a variant-mediated CBEs at 17 sites in HEK293T cells with individual PAMs. Related to **b**, **c**, and Supplementary Fig. 5. Efficiencies of Cas12a variant-mediated CBEs for canonical TTTV PAM (*n* ≥ 5) (**e**) and non-canonical PAMs (*n* ≥ 2) (**f**) in porcine embryos. **g** Average efficiencies of Cas12a variant-mediated CBEs at 14 sites in porcine embryos with individual PAMs. Related to **e**, **f**, and Supplementary Fig. 6. Values and error bars for **b**, **c**, **e**, and **f** represent the mean and SEM, respectively. For **d** and **g**, the boxplots show the median, first quartile, and third quartile, and whiskers represent the maximum and minimum values. Statistical significance was calculated by unpaired two-tailed Mann–Whitney test. ns (not significant), *p* ≥ 0.05; *p < 0.05; **p < 0.01; ***p < 0.001; ****p < 0.0001.

Subsequently, we constructed PAM-expanded CBEs by fusing hA3A-Y130F with the three dCas12a variants and tested them at sites with canonical or non-canonical PAMs in HEK293T cells (Fig. 1a; Supplementary Fig. 4a, b). All four CBEs, including dCas12a-A3A-Y130F, enCas12a-A3A-Y130F, RR-A3A-Y130F, and RVR-A3A-Y130F, can convert C-to-T at TTTV PAM sequences with comparable efficiencies (18.3–50.0%), although the efficiencies of enCas12a-A3A-Y130F were ~1.6-fold higher than those of the other three ones at the *ADAM6* site (Fig. 1b, d; Supplementary Data 1; Supplementary Fig. 5a). However, among the 14 tested sites with non-canonical PAMs, dCas12a-A3A-Y130F showed a poor performance except at two sites, *KLF4-6* and *DNMT1-1*, with VTTV PAM sequences (Fig. 1c, d; Supplementary Data 1; Supplementary Figs. 5a, b), consistent with previous reports^9,19^. By contrast, three engineered Cas12a-variant versions of CBEs showed a robust editing effect when targeting sites with non-canonical PAMs, while the editing efficiencies differed for individual sites. In general, enCas12a-A3A-Y130F exhibited robust editing for most tested sites with VTTV, TTCN, TCCV, TATV, and TTTT PAMs, with editing efficiencies 1.6–2.3-fold that of dCas12a-A3A-Y130F (Fig. 1c, d; Supplementary Data 1; Supplementary Fig. 5a, b). Notably, RR-A3A-Y130F and RVR-A3A-Y130F showed preference in editing sites with TTCN/TCCV (2.4-fold of dCas12a-A3A-Y130F) and TATV (2.3-fold of dCas12a-A3A-Y130F) PAMs, respectively (Fig. 1c, d; Supplementary Data 1; Supplementary Fig. 5a, b). Despite the differences in Cas12a variants adopted, the editing windows of these CBEs spanned ~7 nt (positions 6–12), consistent with that of dCas12a-A3A-Y130F (Supplementary Fig. 5c). These results indicated that these engineered Cas12a variant-derived CBEs retained their capacities for non-canonical PAM recognition.

We thus further tested these CBEs in porcine embryos and observed that dCas12a-A3A-Y130F and its three variants (enCas12a-, RR-, and RVR-A3A-Y130F) can function efficiently at sites with TTTV PAM sequences, with efficiencies of 36.0% to 73.3% (Fig. 1e, g; Supplementary Data 1; Supplementary Fig. 6a). Meanwhile, enCas12a-, RR-, and RVR-A3A-Y130F can recognize and edit sites with the corresponding non-canonical PAMs, where dCas12a-A3A-Y130F showed extremely low efficiencies (Fig. 1f, g; Supplementary Data 1; Supplementary Fig. 6a), consistent with the findings in HEK293T. We observed that enCas12a-A3A-Y130F outperformed the other two engineered variants (RR- and RVR-A3A-Y130F) when targeting sites with VTTV PAM sequences in embryos, with efficiencies of 23.8% vs 4.6 and 10.3%, respectively (Fig. 1f, g; Supplementary Data 1). Although RR- and enCas12a-A3A-Y130F can both edit sites with TTCN/TCCV PAMs, the former showed a higher efficiency for TCCV PAM sequences (Fig. 1f, g; Supplementary Data 1). Similarly, RVR-A3A-Y130F showed the highest efficiencies (21.5%) among four CBEs for TATV/TGTV PAMs (Fig. 1f, g; Supplementary Data 1). The activity window (positions 7–12) in embryos was slightly narrower than that in HEK293T cells (Supplementary Figs. 5c and 6b).

We also fused another hA3A variant (hA3A-N57G) to the dCas12a variants (Supplementary Fig. 4a, b). The results indicated enCas12a-, RR-, and RVR-A3A-N57G retained their preference for TCR and TCCR motifs at sites with canonical TTTV PAM or non-canonical PAMs in HEK293T cells and porcine embryos (Supplementary Fig. 7), which was beneficial to minimizing bystander activities.

These results indicated that CBEs consisting of fused hA3A-Y130F and Cas12a variants, including enCas12a, RR, and RVR, can conduct C-to-T conversion at sites with canonical and non-canonical PAMs efficiently, including NTTN, TYCN, and TRTV, collectively. In addition, the hA3A-N57G variant in these Cas12a variant-mediated CBEs conferred similar TCR/TCCR specificity to the original dCas12a-A3A-N57G.

### Efficient A-to-G conversion by Cas12a variant-mediated ABEs for non-canonical PAMs

To perform A-to-G conversion, we firstly compared two dCas12a-ABEs fused with individual adenine deaminases, including TadA-TadA*7.10^3^ and TadA*8e^23^ respectively, in HEK293T cells and porcine embryos at sites with TTTV PAM sequences (Supplementary Fig. 8a). The results showed that dCas12a-ABE8e with TadA*8e monomer showed considerably more robust efficiency than dCas12a-ABE7, which fused with TadA-TadA*7.10 dimer, although both ABEs showed similar sequence preferences for TA motif (Supplementary Figs. 8, 9), consistent with the findings of a previous study on plants^28^. In addition, a V106W mutation was introduced into TadA*8e, which was reported to reduce RNA off-target^23,29^, and the resulting dCas12a-ABE8e-V106W retained a high efficiency for A-to-G conversion (Supplementary Figs. 8, 9), although the RNA off-target was not evaluated in this study. A similar editing window (positions 8–12) for dCas12a-ABE7, dCas12a-ABE8e, and dCas12a-ABE8e-V106W was observed between the HEK293T cells and porcine embryos (Supplementary Figs. 8e, 9c).

**Fig. 2:**
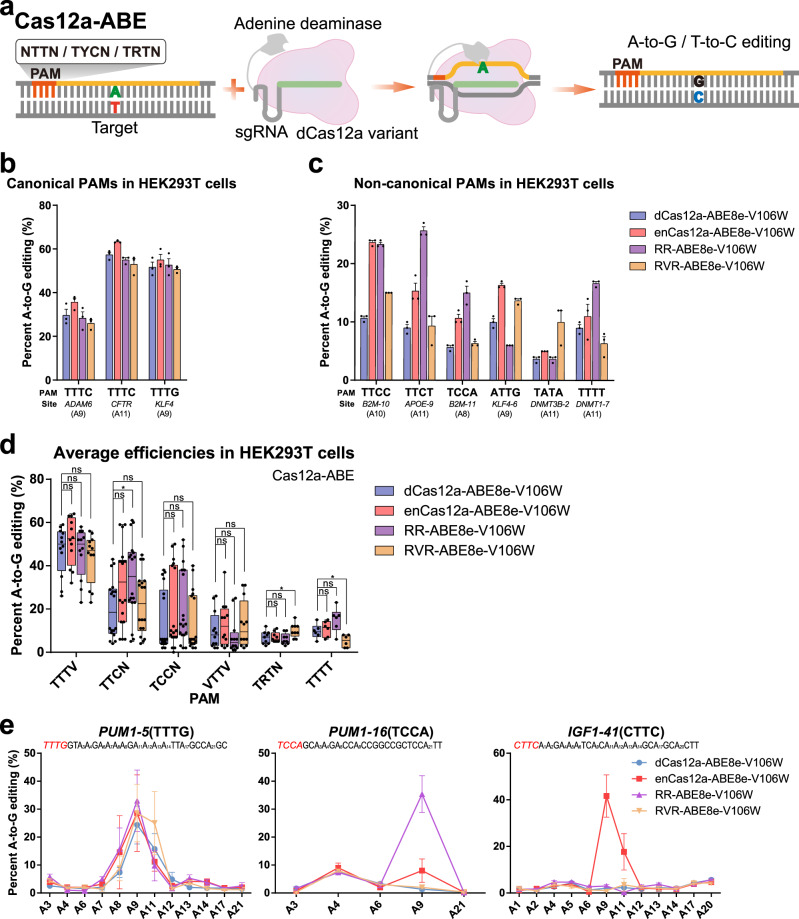
Efficient A-to-G conversion by Cas12a variant-mediated ABEs for non-canonical PAMs. **a** Schematic of A-to-G editing on targets with non-canonical PAMs by Cas12a variant-mediated ABEs. Efficiencies of Cas12a variant-mediated ABEs, including dCas12a-, enCas12a-, RR-, and RVR-ABE8e-V106W, for canonical TTTV PAM (**b**) and non-canonical PAMs (**c**) in HKE293T cells (*n* = 3). **d** Average efficiencies of Cas12a variant-mediated ABEs at 14 sites in HEK293T cells with individual PAMs. Related to **b**, **c**, and Supplementary Fig. 10. **e** Efficiencies of Cas12a variant-mediated ABEs for canonical TTTV PAM and non-canonical PAMs, TCCA and CTTC, in porcine embryos (*n* ≥ 3). Values and error bars for **b**, **c**, and **e** represent the mean and SEM, respectively. For **d**, the boxplots show the median, first quartile, and third quartile, and whiskers represent the maximum and minimum values. Statistical significance was calculated by unpaired two-tailed Mann–Whitney test. ns (not significant), *p* ≥ 0.05; *p < 0.05.

We thus attempted to construct new ABEs with expanded target scope by fusing engineered Cas12a variants with TadA*8e-V106W, resulting enCas12a-ABE8e-V106W, RR-ABE8e-V106W, and RVR-ABE8e-V106W, which were expected to convert A-to-G at sites with non-canonical PAMs (Fig. 2a; Supplementary Figs. 4a, c).

We then tested the base editing efficiencies and preferences at endogenous sites with TTTV PAM or other non-canonical PAMs in HEK293T cells. These Cas12a variant-derived ABEs performed efficient A-to-G editing at sites with non-canonical PAMs, in addition to sites with TTTV PAM sequences (Fig. 2b–d; Supplementary Data 1; Supplementary Fig. 10). Among the three ABE variants, enCas12a-ABE8e-V106W showed robust editing activities at sites with TTCC (23.7%) and ATTG (16.3%) PAMs, which were 2.2-fold and 1.6-fold that of dCas12a-ABE8e-V106W, respectively (Fig. 2c; Supplementary Data 1). RR-ABE8e-V106W exhibited efficient A-to-G editing for TTCC/TTCT/TCCA/TTTT PAMs (20.1% on average), whereas RVR-ABE8e-V106W can edit sites with TATA PAM sequences, with frequencies increasing by twofold compared with those of other ABEs (Fig. 2c; Supplementary Data 1). For several tested non-canonical PAMs, the efficiency differences were not as drastic as expected among individual Cas12a variants (Supplementary Fig. 10b), which disagrees with previous reports^17,18^, and the cause was unknown.

We also confirmed the capacities of these new ABEs for alternative PAM recognition in porcine embryos at three sites with canonical or non-canonical PAMs. All four ABEs showed comparable efficiencies for TTTG PAMs (24.3–33.0%), whereas RR-ABE8e-V106W and enCas12a-ABE8e-V106W exhibited the highest efficiencies for TCCA (35.4%) and CTTC (41.7%) PAMs, respectively (Fig. 2e; Supplementary Data 1).

These results revealed that the Cas12a variant-derived ABEs fused with TadA*8e-V106W can alleviate the limit of wild-type Cas12a for TTTV PAM recognition and execute A-to-G conversions at TTTV and alternative PAMs in mammalian cells and embryos.

### Multiplexed base editing by Cas12a variant-mediated CBEs and ABEs

**Fig. 3:**
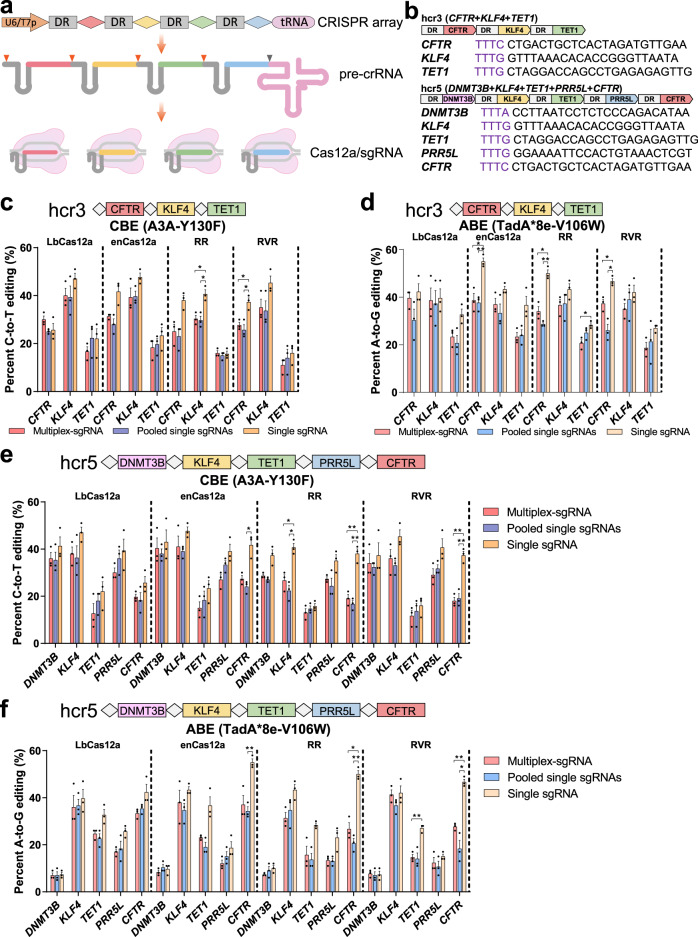
Multiplexed base editing by Cas12a variant-mediated base editors in HEK293T cells. **a** Schematic illustrating a single CRISPR array processed by Cas12a per se to generate multiple mature sgRNAs for simultaneous multiple loci editing. A tRNA precursor sequence appended to downstream of the array is transcribed along with pre-crRNA and cut by RNase P as previously reported^51^. Rhombuses with distinctive colors indicate individual spacers. Orange and gray triangles denote the cleavage sites of Cas12a and RNase P, respectively. U6/T7p U6 or T7 promoter; DR direct repeat sequence; tRNA transfer RNA. **b** Constructs of CRISPR arrays of hcr3 with three tandem sgRNAs and hcr5 with five tandem sgRNAs targeting to human genome. The sequences of individual targets are listed below, and PAMs are marked in purple. Simultaneous base editing of three loci (*CFTR*, *KLF4*, and *TET1*) in HEK293T cells by Cas12a variant-mediated CBEs (**c**) and ABEs (**d**) with multiplex-sgRNA (CRISPR array hcr3) or pooled single sgRNAs, with a single sgRNA for a single locus as the control (*n* = 3). Simultaneous base editing of five loci (*DNMT3B*, *KLF4*, *TET1*, *PRR5L*, and *CFTR*) in HEK293T cells by Cas12a variant-mediated CBEs (**e**) and ABEs (**f**) with multiplex-sgRNA (CRISPR array hcr5) or pooled single sgRNAs, with a single sgRNA for a single locus as the control (*n* = 3). Values and error bars for **c**–**f** represent the mean and SEM, respectively. Statistical significance was calculated by Welch’s ANOVA test, and Tamhane’s T2 multiple comparisons test was performed. ns (not significant, *p* ≥ 0.05) is not shown, and only *p* < 0.05 is shown (*p < 0.05, **p < 0.01).

To take advantage of the unique feature of Cas12a in multiplexed genome editing, we harnessed Cas12a variant-mediated CBEs and ABEs to perform multiplexed base editing, simultaneously, at several loci with a single CRISPR array (termed as multiplex-sgRNA) (Fig. 3a; Supplementary Fig. 11).

We initially tested in HEK293T cells with a multiplex-sgRNA containing three tandem sgRNAs (named hcr3) to edit three sites with TTTV PAM sequences simultaneously (Fig. 3b). For the control, we targeted three sites with pooled single sgRNAs and a single site with the corresponding sgRNA in parallel (Supplementary Fig. 11). All three sites, including *CFTR*, *KLF4*, and *TET1*, can be edited effectively by these CBEs and ABEs with hcr3, and the efficiencies were comparable or superior to those with pooled sgRNAs (Fig. 3c, d; Supplementary Data 1). For example, dCas12a-A3A-Y130F showed an efficiency of 30.0% at *CFTR* with hcr3 compared with 25.0% observed with pooled sgRNAs (Fig. 3c; Supplementary Data 1). Similarly, RVR-ABE8e-V106W exhibited an improved efficiency with hcr3 compared with pooled sgRNAs at *CFTR* (37.3% *vs* 26.0%) (Fig. 3d; Supplementary Data 1). Both the tandem multiplex-sgRNA and pooled sgRNAs contributed compromised efficiencies of C-to-T and A-to-G editing, compared with single sites edited alone. The average efficiencies of four CBEs for *CFTR*, *KLF4*, and *TET1* with hcr3 were 28.5, 36.2, and 15.5%, respectively (Fig. 3c; Supplementary Data 1). By contrast, the corresponding efficiencies were 35.7, 45.2, and 19.3%, respectively, when a single site was targeted with a single sgRNA (Fig. 3c; Supplementary Data 1). Similar results were observed in multiplexed editing using Cas12a-ABEs (Fig. 3d; Supplementary Data 1). The relative inefficiency of multiple editing, when compared with a single locus, was consistent with the previous observations^12,15,30^. This finding may be due to the unsaturated base editor proteins at individual sites. As in our transfection experiments, the amount of expression plasmids for the base editor proteins remained constant although the number of target sites increased.

Further, we attempted to extend the number of simultaneous base editing loci to five with tandem multiplex-sgRNA (named hcr5) (Fig. 3b), and observed that all the five endogenous sites, including *DNMT3B*, *KLF4*, *TET1*, *PRR5L*, and *CFTR*, can be edited by these Cas12a variant-mediated CBEs and ABEs (Fig. 3e, f; Supplementary Data 1). The efficiencies of C-to-T editing for enCas12a-A3A-Y130F at the five sites were 40.3, 41.0, 15.0, 27.0, and 27.3%, respectively (Fig. 3e; Supplementary Data 1). Accordingly, the efficiencies of A-to-G editing for enCas12a-ABE8e-V106W were 8.3, 38.0, 23.0, 12.0, and 37.0%, respectively (Fig. 3f; Supplementary Data 1). For the *DNMT3B* site, a low efficiency for ABEs was due to all As located outside the main activity window.

We observed comparable efficiencies for the *CFTR* site between CRISPR array hcr3 and hcr5, where the *CFTR*-sgRNA is located at the first and fifth positions, respectively (Fig. 3c–f; Supplementary Data 1; Supplementary Fig. 12). This result indicated, to a certain extent, that positioning within the array is not crucial for efficiency variation but likely the target itself, consistent with those of previous reports^11,13,14^.

**Fig. 4:**
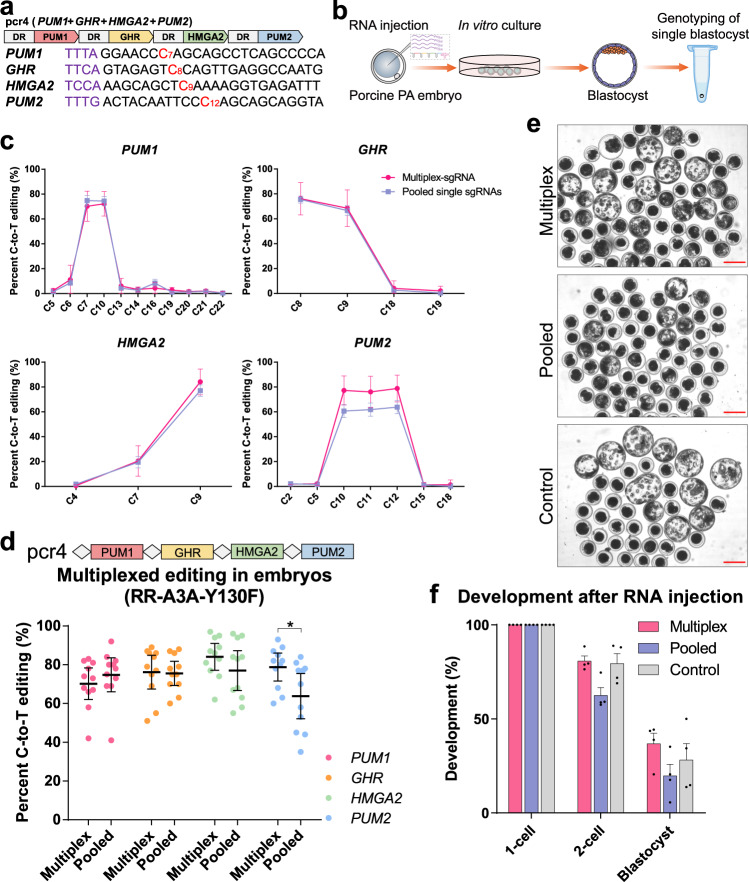
Multiplexed base editing by Cas12a variant-mediated base editors in embryos. **a** Construct of CRISPR array of pcr4 with four tandem sgRNAs targeting to porcine genome. The sequences of individual targets are listed below. PAMs are marked in purple and target Cs are marked in red. **b** Schematic illustrating the workflow of RNA mixture microinjection of porcine parthenogenetic (PA) embryos to assess the editing efficiency. **c** Simultaneous C-to-T editing of four loci (*PUM1*, *GHR*, *HMGA2*, and *PUM2*) by RR-A3A-Y130F with multiplex-sgRNA (CRISPR array pcr4) or pooled single sgRNAs in porcine PA embryos (*n* = 11). **d** Efficiencies of C-to-T editing of target Cs (marked in red in **a**) induced by RR-A3A-Y130F at four indicated loci (*n* = 11). **e** Representative images of porcine PA embryos development in vitro six days after RNA injection, with uninjected PA embryos as the control (*n* = 4). Scale bar, 200 µm. **f** Development of porcine PA embryos in vitro after RNA injection (*n* = 4). Values and error bars for **c** and **f** represent the mean and SEM, respectively. Values for **d** represent the median with interquartile range. Statistical significance was calculated by unpaired two-tailed Student’s *t*-test (*p < 0.05).

Given the positive results from HEK293T cells, we examined the efficiencies of multi-loci base editing with a single CRISPR array in embryos, especially for sites with non-canonical PAMs. Four genes, including *PUM1*, *GHR*, *HMGA2*, and *PUM2*, in porcine genome were thus selected as targets (Fig. 4a), which are all associated with body sizes. Premature stop codons were expected to be generated through C-to-T conversion by Cas12a-CBE. Particularly, *PUM1* and *PUM2* possessed canonical TTTV PAM, whereas non-canonical TTCA and TCCA for *GHR* and *HMGA2*, respectively (Fig. 4a). In view of the inclusion of TTCA/TCCA PAMs, we employed RR-A3A-Y130F, a variant that recognize TYCV PAM sequence (Y = C or T), along with tandem multiplex-sgRNA (named pcr4) or pooled sgRNAs in porcine parthenogenetic (PA) embryos by microinjection (Fig. 4b). The results showed that the pcr4 can attain high efficiencies of 70.2, 76.2, 84.1, and 78.8% on average for *PUM1* (C7), *GHR* (C8), *HMGA2* (C9), and *PUM2* (C12), respectively (Fig. 4c, d; Supplementary Data 1). Pooled sgRNAs resulted in considerable editing efficiencies although the values were slightly lower for *PUM2*. However, the pooled sgRNAs showed more variability among individual embryos for the four sites compared with the multiplex-sgRNA (Supplementary Fig. 13). Importantly, the pooled sgRNAs probably impaired the embryonic development to a certain extent (Fig. 4e, f; Supplementary Data 1), presumably due to the increased amount of the injected RNAs.

These results indicated that multiplexed base editing with up to five and four loci in HEK293T cells and embryos, respectively, can be realized by a single CRISPR array along with these Cas12a variant-derived CBEs and ABEs. Compared with pooled sgRNAs, a single transcript of CRISPR array can potentially maintain a considerable transfection efficiency in mammalian cells and minimize the adverse effect of microinjection when multiple loci need to be modified.

### Product purity and off-target analysis of Cas12a variant-mediated base editors

Finally, we evaluated the editing precision of these Cas12a variant-mediated base editors, including the product purity, level of indels, and off-target activity. Sites with canonical TTTV PAM sequences in human genome were selected to avoid the preference of Cas12a variants for distinct PAMs (Supplementary Data 2 and 3). Also, a non-targeting sgRNA (scrambled sgRNA), which contains the same nucleotide composition as *CFTR* while does not target the human genome, was used as a negative control (Supplementary Data 2).

**Fig. 5:**
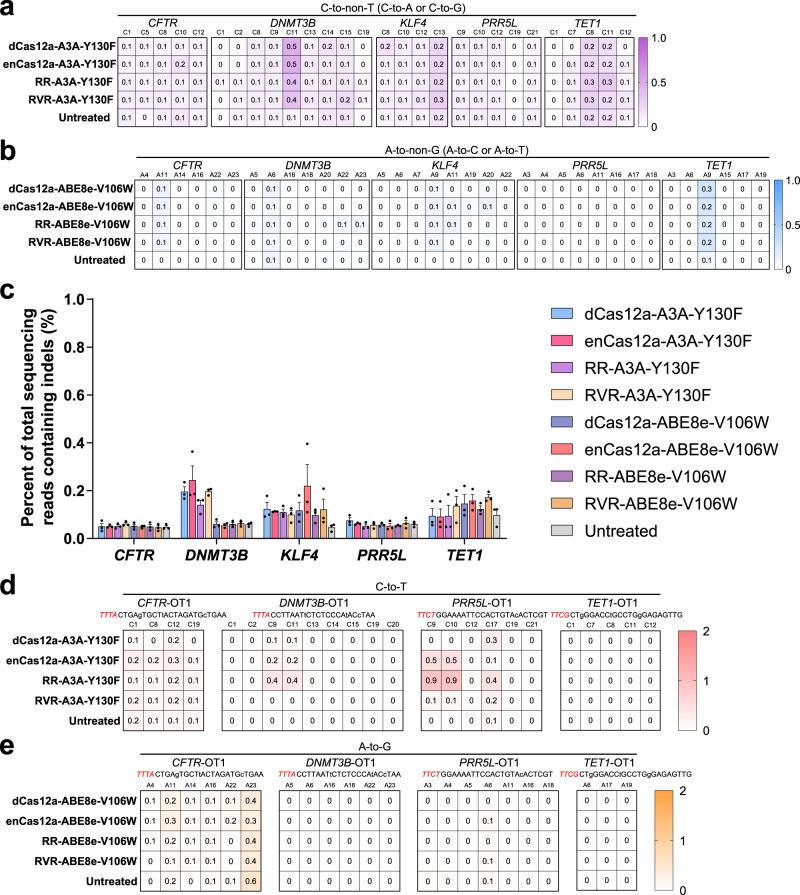
Specificity of Cas12a variant-mediated base editors in HEK293T cells. Heat maps showing the frequencies of C-to-non-T (C-to A or C-to-G) conversion for CBEs (**a**) and A-to-non-G (A-to-C or A-to-T) for ABEs (**b**) at five selected sites with TTTV PAM (*n* = 3). **c** Indels frequencies for indicated CBEs and ABEs at the tested sites (*n* = 3). Heat maps showing the Cas12a-dependent off-target activity of indicated CBEs (**d**) and ABEs (**e**) at the predicted off-target sites (*n* = 3). The spacer sequences of off-target sites are shown with mismatches marked in lowercase and PAM marked in red and in italics. Values and error bars represent the mean and SEM, respectively. All heatmaps show the mean of three independent biological replicates.

Targeted deep sequencing of the selected targets showed that Cas12a and its variants, including enCas12a, RR, and RVR, resulted in a high product purity, with slightly unintended C-to-non-T editing for CBEs and A-to-non-G for ABEs, respectively (Fig. 5a, b; Supplementary Data 1; Supplementary Figs. 14, 15a, b). Notably, indels can only be detected at the background level for all these Cas12a variant-mediated base editors (Fig. 5c; Supplementary Data 1; Supplementary Fig. 15c), which can be attributed to the use of catalytically dead Cas12a instead of nickase ones, consistent with the previous study^21^.

To evaluate the off-target activity of the developed base editors, we considered Cas12a-dependent off-target from the tolerance for mismatch, which represented the most dominant part of the off-target effect^31^. Potential off-target sites were predicted using the Cas-OFFinder^32^ tool (http://www.rgenome.net/cas-offinder/), with up to three nucleotide mismatches and PAMs restricted to NTTN, TYCN and TRTN (Supplementary Data 3). Among these potential off-target sites, most showed no evident off-target editing (Fig. 5d, e; Supplementary Data 1; Supplementary Figs. 16–18), except for *PRR5L*-OT1 and *PRR5L*-OT3, which shared one and two mismatches with the *PRR5L* target, respectively (Fig. 5d; Supplementary Data 1; Supplementary Fig. 16). Consistent with the relaxed PAM compatibility^17–19^, off-target editing for *PRR5L*-OT1 and *PRR5L*-OT3, both with TTCT PAM sequences, occurred only for base editors derived from enCas12a and RR but not for those from dCas12a and RVR (Fig. 5d; Supplementary Data 1; Supplementary Fig. 16). Cas12a-independent DNA and RNA off-target effect, which was mainly attributed to the nature of deaminases, has not been detected in this study. We speculated a substantial mitigation for these CBEs and ABEs when Y130F or N57G in hA3A and V106W in TadA*8e were introduced, respectively, as demonstrated by several previous studies in detail^21,23,27,29,33–38^.

These results indicated that the established Cas12a variant-mediated CBEs and ABEs displayed a considerably adequate precision and specificity at the tested sites with high product purity, negligible indels, and low off-target activity (Table 1; Supplementary Data 1).

**Table 1:** Summary of Cas12a variant-mediated CBEs and ABEs in this study.

Base editor	Cas12a variant	Deaminase	Motif preference	PAM	Activity window	Editing efficiency		Specificity = (on–off) /on
						HEK293T cells	Embryos	
dCas12a-A3A-Y130F	dLbCas12a wild-type	hAPOBEC3A (Y130F)	No	TTTN	~6–12	51.0%	73.3%	99.8%
enCas12a-A3A-Y130F	enCas12a dead			NTTN + TTCN + TRTV	~6–12	55.7%	71.1%	98.9%
RR-A3A-Y130F	RR dead			TTTN + TYCN	~6–12	50.0%	63.5%	98.0%
RVR-A3A-Y130F	RVR dead			TTTN + TRTV	~6–12	44.7%	51.9%	99.8%
dCas12a-A3A-N57G	dLbCas12a wild-type	hAPOBEC3A (N57G)	TCR/TCCR	TTTN	~7–11	39.3%	60.5%	n.d.
enCas12a-A3A-N57G	enCas12a dead			NTTN + TTCN + TRTV	~7–11	42.7%	75.3%	n.d.
RR-A3A-N57G	RR dead			TTTN + TYCN	~7–11	42.3%	80.0%	n.d.
RVR-A3A-N57G	RVR dead			TTTN + TRTV	~7–11	32.0%	62.4%	n.d.
dCas12a-ABE8e-V106W	dLbCas12a wild-type	TadA*8e (V106W)	TA > CA > GA > AA	TTTN	~8–12	57.3%	24.3%	99.6%
enCas12a-ABE8e-V106W	enCas12a dead			NTTN + TTCN + TRTV	~8–12	63.3%	41.7%	99.6%
RR-ABE8e-V106W	RR dead			TTTN + TYCN	~8–12	60.0%	35.4%	99.6%
RVR-ABE8e-V106W	RVR dead			TTTN + TRTV	~8–12	53.0%	28.6%	99.6%

Editing efficiency indicated the highest efficiency of tested sites in this study (related to Figs. 1 and 2; Supplementary Figs. 5–7 and 10). Specificity was calculated as (*E*^on^ − *E*^off^)/*E*^on^, where *E*^on^ represents the highest editing efficiency of the target site and *E*^off^ represents the highest editing efficiency of the off-target site, and the values shown indicated the average of four sites (*DNMT3B*, *TET1*, *PRR5L*, and *CFTR*). *n.d.* not detected.

## Discussion

Base editors have been developed to perform precise genome modification with high efficiency and specificity, and they are powerful for the precise base substitution of single or multiple loci^1,3,6,39–41^. Without inducing double-stranded DNA breaks (DSBs), base editors also show significant advantages in multi-gene disruption by introducing premature stop codons at multiple loci, whereas traditional nucleases mediate DSBs at multiple loci, possibly increasing the risk of genomic instability and chromosomal variation^42,43^. Despite their superiority, base editors have only been harnessed for multiplexed editing by pooling multiple sgRNAs^40,41^, which is potentially compromised by the transfection efficiencies in cultured cells and toxic effects in embryos with the increase in sgRNAs. Transfer RNA (tRNA) was recently adopted in the tandem sgRNAs of Cas9 system to achieve multiplexed base editing^44,45^. However, this strategy complicates its construction and application and may also be compromised by the insufficient transcription of sgRNAs. By contrast, Cas12a enables multiplexed targeting with a single CRISPR array^10–15^, which is also promising in multiplexed base editing. In this study, we have engineered new Cas12a-mediated CBEs and ABEs with high efficiencies and alleviative PAM limitations by combining highly active hA3A or TadA*8e with the indicated dCas12a variants (Table 1; Supplementary Data 1) and verified that these versatile Cas12a variant-derived base editors can engage in multiplexed base editing with a single transcript of CRISPR array in mammalian cells and embryos.

We have only tested multiplexed editing up to five loci with the tandem CRISPR array. However, extending to a greater number of loci can be reasonably expected as shown in previous report^14^. The reduction in the number of plasmids or RNA will potentially improve the transfection efficiencies in mammalian cells and minimize the adverse effects of microinjection on embryos while retaining intrinsic efficiencies of base editing. Meanwhile, the protein-coding sequence of base editors and tandem CRISPR array can be encoded in a single transcript^14^. This all-in-one strategy shows as a unique superiority for Cas12a while it is difficult to be used in the Cas9 system, which highlights the irreplaceability of Cas12a-mediated base editor systems for multiplexed base editing. Our limited results showed no evident position effect of sgRNAs within the array, which is supported by previous studies^11,13,14^. However, this statement has not been validated in this research and the expression of individual sgRNAs from CRISPR array has not been detected. Thus, more comprehensive pieces of evidence are needed.

In addition to the enCas12a, RR, and RVR variants adopted in this study that collectively recognize NTTN, TYCN, and TRTV PAMs, the LbCas12a variant, that is, impLbCas12a^19^, has recently been reported through the combination of the mutations of RR, RVR, and enCas12a variants, which showed a preference for TNTN PAM and partly overlapped with enCas12a. However, existing Cas12a variants are insufficient for researchers to target almost all interested sites, especially for non-canonical PAMs. Therefore, more efforts and strategies are essential to further loosen the PAM constraint of Cas12a to exploit fully its advantages in multiplexed base editing.

Notably, Cas12a-mediated base editors may be less efficient than Cas9 in certain genome loci although a significant improvement has been achieved compared with the original version. This relative inefficiency is most likely due to the employment of dCas12a, instead of Cas12a nickase, which is not available at the present^1,6,16^, given that previous studies have indicated nicking the unedited strand can improve the base conversion efficiencies^1–3^. Alternatively, the efficiencies of Cas12a-mediated base editors can potentially be elevated further through strategies other than the deaminase activity, such as employing dCas9 to auxiliarily bind the proximity location of the target^20^, exploiting more robust Cas12a variants^15,46^, or covalent conjugating crRNA to Cas12a^47^. The adoption of dCas12a in base editors, on the other hand, offers application benefits, such as low DNA damage response and reduced indels^21^.

We have only analyzed Cas12a-depedent off-target effects of these base editors but not Cas12a-independent off-target effects on the genome and transcriptome^29,31,34–37^. Nevertheless, a comprehensive assessment of the off-target effects is valuable in the future to boost their application.

In conclusion, we have engineered a series of Cas12a-mediated CBEs and ABEs with improved efficiencies and targeting scope by adopting highly active deaminases and Cas12a variants. These base editors have shown dramatically elevated efficiencies at sites with non-canonical PAMs. Notably, leveraging the dual RNase/DNase function of Cas12a, simultaneous base editing of multiple loci with a single CRISPR array can be realized flexibly through these CBEs and ABEs. The established base editors will serve as valuable tools for multiplexed base editing and substantially promote their wide applications in agriculture and biomedicine.

## Methods

### Plasmid construction

Vector dCas12a-A3A was constructed previously^20^ by replacing rAPOBEC1 with an hAPOBEC3A fragment in restriction endonuclease-linearized dCas12a-BE expression vector using the ClonExpress MultiS kit (Vazyme). dCas12a-A3B, dCas12a-A3G, dCas12a-CDA1, dCas12a-AID, and dCas12a-eAID expression vectors were constructed using a similar strategy. To generate fragments of enCas12a, RR, and RVR, we used dLbCas12a to introduce D156R/G532R/K538R, G532R/K595R, and G532R/K538V/Y542R mutations, respectively, by polymerase chain reaction (PCR) amplification with the corresponding primers. These fragments of Cas12a variants were then cloned into restriction endonuclease-linearized dCas12a-A3A-Y130F or dCas12a-A3A-N57G to replace the original dLbCas12a, resulting in the construction of enCas12a-A3A-Y130F, RR-A3A-Y130F, RVR-A3A-Y130F, enCas12a-A3A-N57G, RR-A3A-N57G, and RVR-A3A-N57G expression vectors.

dCas12a-ABE7 expression vector was constructed by cloning dCas12a fragment into the linearized Cas9-ABE using the ClonExpress MultiS kit (Vazyme). The TadA*8e fragment was amplified from TadA*7.10 in Cas9-ABE by several pair of primers to introduce eight mutations (A109S/T111R/D119N/H122N/Y147D/F149Y/T166I/D167N), and an additional V106W mutation was introduced to generate the TadA*8e-V106W fragment. Then, dCas12a-ABE8e and dCas12a-ABE8e-V106W expression vectors were constructed by cloning TadA*8e and TadA*8e-V106W fragments into the linearized dCas12a-ABE7 vector to replace the TadA-TadA*7.10 fragment, respectively. Expression vectors enCas12a-ABE8e-V106W, RR-ABE8e-V106W, and RVR-ABE8e-V106W were constructed by cloning three Cas12a variants into linearized dCas12a-ABE8e-V106W to replace dCas12a.

Target-specific oligonucleotides were annealed and ligated into BpiI-linearized U6-sgRNA plasmid to generate corresponding sgRNA expression vectors. Target sequences were listed in Supplementary Data 2.

### HEK293T cell culture, transfection, and sorting

HEK293T cells (obtained from ATCC and preserved by our laboratory) were maintained in Dulbecco’s Modified Eagle Medium high glucose (HyClone) supplemented with 10% fetal bovine serum (Gibco). Cells were seeded in a 24-well plate at a density of approximately 1.6 × 10^5^ cells per well to reach 70–90% confluence and transfected with 50 µL serum-free Opti-MEM (Gibco), which contained 3 µg polyethylenimine (PEI) (Sigma-Aldrich), 0.3 µg sgRNA expression vector and 0.7 µg base editor expression vector. Cells transfected with 50 µL serum-free Opti-MEM, which only contained 3 µg PEI (without vector), were served as untreated control. About 60 h after transfection, EGFP^+^ cells were sorted by FACS AriaIIU (Becton Dickinson), given that all base editor expression vectors contain EGFP fluorescence labels, and subjected to lysis in 10 µL lysis buffer with 1% NP40 (MP Biomedicals) and 50 µg mL^−1^ proteinase K (Tiangen).

### In vitro transcription

MssI-linearized base editor vectors, including the T7 promoter, were used as templates to transcribe mRNA in vitro using the HiScribe T7 ARCA mRNA Kit (with tailing) (New England Biolabs), and mRNA was purified using the RNeasy MiniElute Cleanup Kit (Qiagen) in accordance with the manufacturer’s instructions. To produce sgRNAs, we amplified templates from the corresponding U6-sgRNA vectors, accompanied by the introduction of T7 promoter sequence, and then transcribed them using the HiScribe T7 Quick High Yield RNA Synthesis Kit (New England Biolabs). Transcribed sgRNAs were purified using the RNeasy MiniElute Cleanup Kit (Qiagen).

### Porcine embryo microinjection and culture in vitro

Porcine PA embryos were used in our microinjection, and oocyte collection, in vitro maturation, and parthenogenetic activation were conducted as previously described^40^. Briefly, immature porcine oocytes, which were derived from ovaries obtained from a slaughterhouse, were matured in vitro. Mature oocytes with visible first polar bodies were then subjected to electrical activation to initiate PA development. Six hours after activation, the one-cell embryos were injected with RNA mixture of the base editor mRNA (150 ng µL^−1^) and sgRNA (50 ng µL^−1^) into the cytoplasm by the FemtoJet5247 microinjector (Eppendorf). The embryos were then cultured in vitro with PZM-3 medium for 6 days, followed by collection of single embryos for lysis in 5 µL lysis buffer.

### Genotyping of HEK293T cells and porcine embryos

Target genomic sites were PCR amplified using the lysate of HEK293T cells and porcine embryos by the Rapid Taq Master Mix (Vazyme), with primers flanking each examined target site. Sanger sequencing was then performed by IGE Biotechnology (Guangzhou, China), and the ratios of C-to-T and A-to-G conversions were calculated by the EditR software^48^ (https://moriaritylab.shinyapps.io/editr_v10/). The primers used to amplify the target genomic sequences are listed in Supplementary Data 2.

### On-target and off‑target analyses

For target deep sequencing, primers containing Illumina forward and reverse adapters were used to amplify genomic regions spanning the target sites for 30 cycles of the first round of PCR using the Phanta Max Super-Fidelity DNA Polymerase (Vazyme), followed a second round with 12 cycles of PCR amplification with barcode-containing Illumina primers. The purified products of the second round of PCR amplicons with different tags were pooled and subjected to Annoroad Biotechnology (Beijing, China) for deep sequencing on the Illumina NovaSeq 6000 platform. The deep sequencing data were analyzed through the CRISPResso2 tool^49^.

Potential off-targets of selected target sites were predicted using the previous reported Cas-OFFinder^32^ (http://www.rgenome.net/cas-offinder/), with up to three mismatches and no bulge, and the PAM sequences were restricted to NTTN, TYCN, and TRTN. Once more than ten potential off-targets for one site were predicted, only ten were analyzed. The amplicons spanning the predicted off-targets with unique barcodes were pooled and used for deep sequencing as described above.

Potential off-target sequences and primers used to amplify the on-target and off-target sites are listed in Supplementary Data 3.

### Statistics and reproducibility

Statistics and graphs were prepared using Prism v8 (GraphPad), and error bars indicated the mean with standard error of the mean (SEM). Statistical significance was determined using the corresponding methods described in figure legends, with *p* values of <0.05 considered statistically significant. Data represented the results of three independent replicate experiments unless mentioned specially, and sample sizes were indicated in figure legends.

### Reporting Summary

Further information on research design is available in the Nature Research Reporting Summary linked to this article.

## Supplementary information

Supplementary Information

Description of Additional Supplementary Data

Supplementary Data 1

Supplementary Data 2

Supplementary Data 3

Reporting Summary-New

## Data Availability

All deep-sequencing data from this study have been deposited in the Genome Sequence Archive of the National Genomics Data Center, China National Center for Bioinformation^50^ (accession number: HRA002437). The source data for Figs. 1–5 and Table 1 can be found in Supplementary Data 1. The expression plasmids are available from addgene (accession IDs: 193638 to 193662). Other materials supporting the findings of this study are available upon reasonable request.
