# Supplementary material for: Multiplexed base editing through Cas12a variant-mediated cytosine and adenine base editors
Source: Commun Biol. 2022 Nov 2;5:1163. doi: 10.1038/s42003-022-04152-8 (PMC9630288; doi:10.1038/s42003-022-04152-8)
Supplement: Supplementary file 1 — Supplementary Information [file 42003_2022_4152_MOESM1_ESM.pdf]

Supplementary Figures

Multiplexed base editing through Cas12a variant-mediated cytosine and adenine base editors

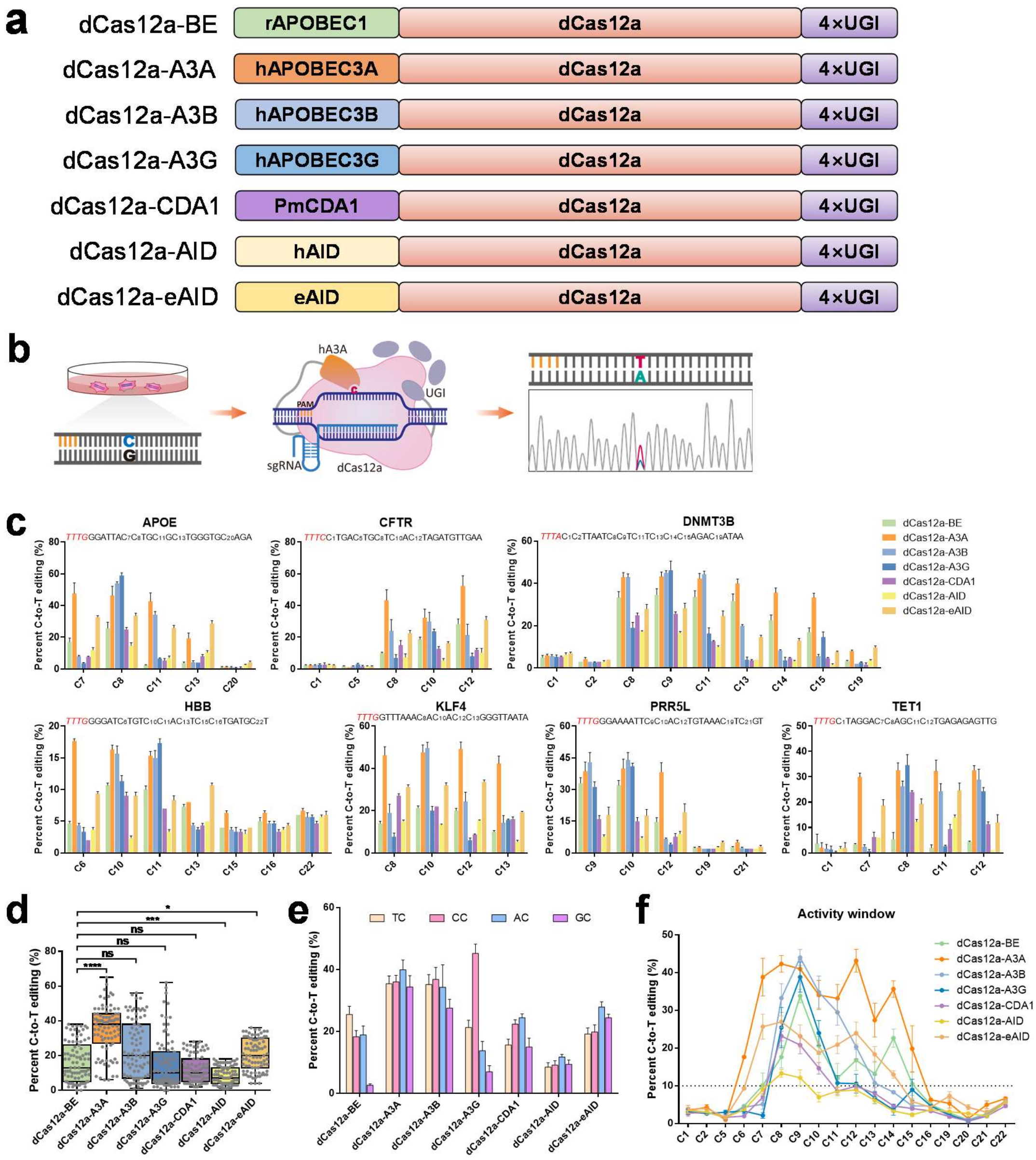

Supplementary Fig. 1 Comparisons of different CBEs with individual cytosine deaminases fused to dCas12a in HEK293T cells.

a Schematic representation of Cas12a-CBEs with different cytosine deaminases fused to the N-terminal and uracil DNA glycosylase inhibitor (UGI) fused to the C-terminal. **b** Schematic illustrating the C-to-T conversion by base editor dCas12a-A3A in cultured cells. **c** C-to-T editing mediated by different Cas12a-CBEs at 7 sites in HEK293T cells (n=3). **d** Average efficiencies of Cas12a-CBEs at 7 sites shown in **c**. **e** Sequence preferences for of different Cas12a-CBEs. **f** Activity windows of different Cas12a-CBEs, with the positions lacking cytosine in all 7 tested sites omitted and 10% as a baseline. Values and error bars for **c**, **e**, and **f** represent the mean and SEM, respectively. For **d**, the boxplots show the median, first quartile, and third quartile, and whiskers represent the maximum and minimum values. Statistical significance was calculated by Kruskal–Wallis test, and Dunn's multiple comparisons test was performed. ns indicates not statistical significant, *$p < 0.05$, ***$p < 0.001$, and ****$p < 0.0001$.

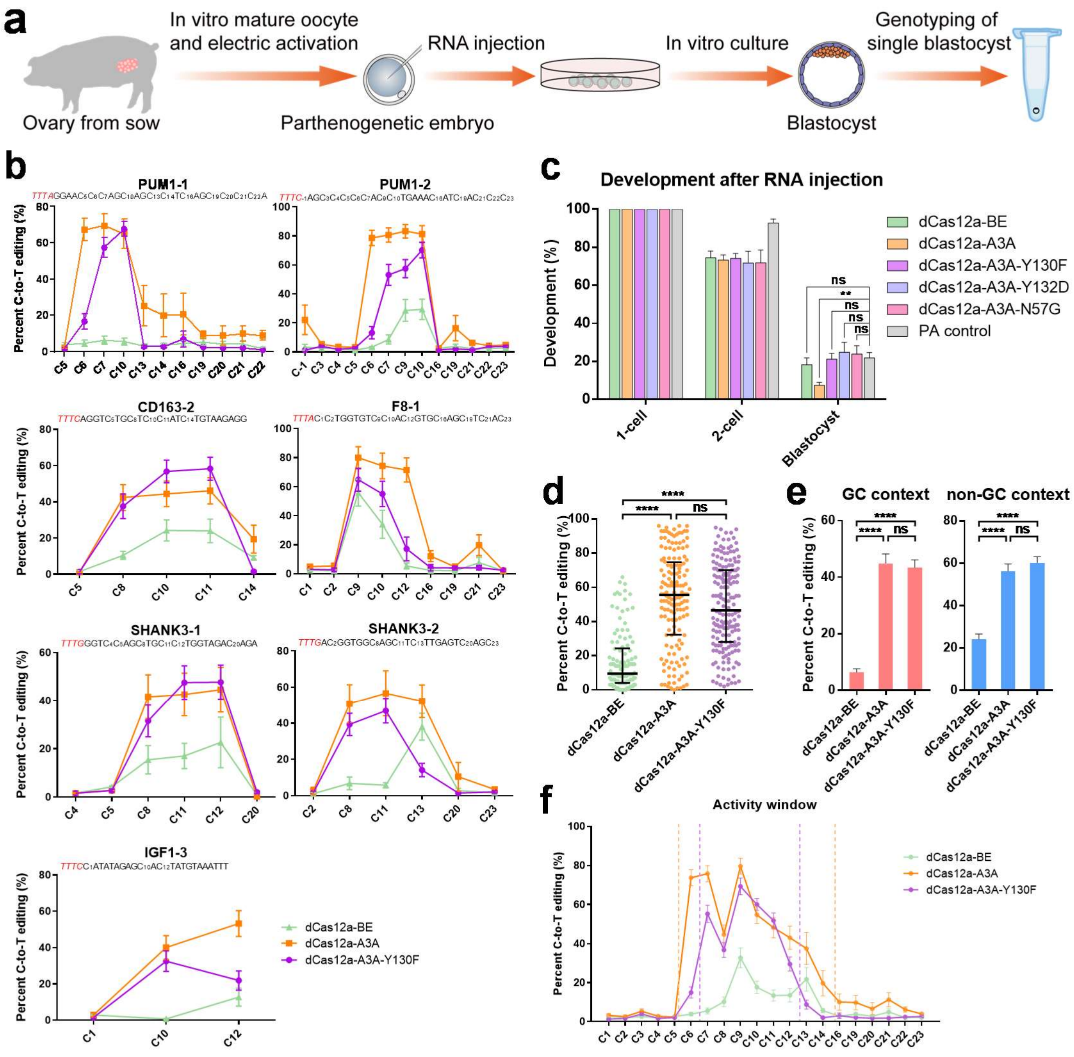

Supplementary Fig. 2 Robust C-to-T conversions mediated by dCas12a-A3A in parthenogenetic (PA) embryos.

a Flow diagram showing the efficiency assessment of base editors in embryos by RNA injection of porcine PA embryos. **b** C-to-T editing mediated by different Cas12a-CBEs at 7 sites in porcine PA embryos (n≥3). **c** Development of porcine PA embryos *in vitro* after RNA injection (n≥4). **d** Average efficiencies of Cas12a-CBEs at 7 sites shown in **b**. **e** Efficiencies of dCas12a-BE, dCas12a-A3A, and dCas12a-A3A-Y130F for GC and non-GC motifs. **f** Activity windows of different Cas12a-CBEs, with the positions lacking cytosine in all 7 tested sites omitted. Values and error bars for **b**, **c**, **e**, and **f** represent the mean and SEM, respectively. Values for **d** represent the median with interquartile range. Statistical significance was calculated by Kruskal–Wallis test, and Dunn's multiple comparisons test was performed. ns indicates not statistical significant, $**p < 0.01$, and $****p < 0.0001$.

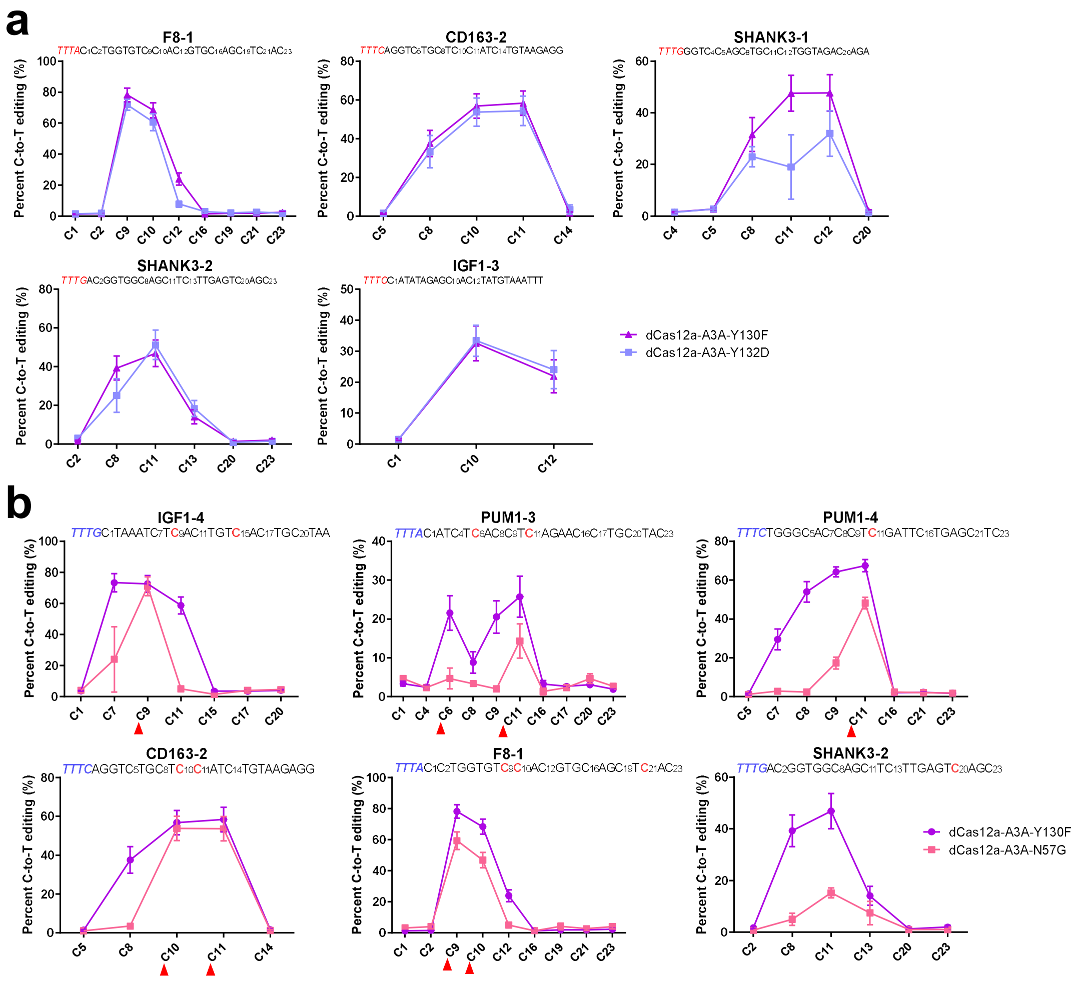

Supplementary Fig. 3 Comparisons of the efficiencies of CBEs with hA3A variants fused to dCas12a in porcine embryos.

a Comparison of C-to-T editing efficiencies executed by dCas12a-A3A-Y130F and dCas12a-A3A-Y132D in embryos (n≥4). **b** Selective C-to-T editing induced by dCas12a-A3A-N57G for TCR/TCCR motifs in embryos, indicated by the red arrowheads (n≥2). Values and error bars represent the mean and SEM, respectively.

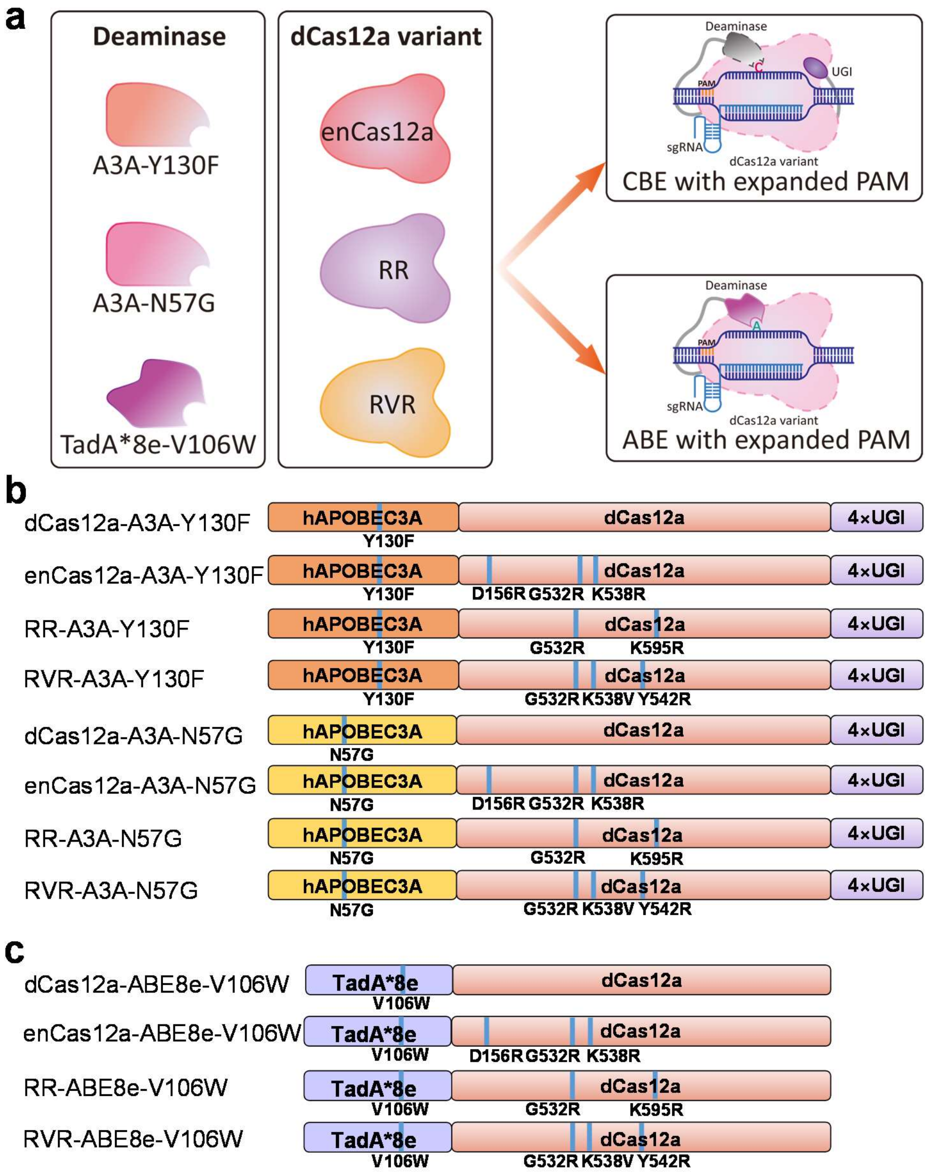

Supplementary Fig. 4 Schematic illustrating the construction of CBEs and ABEs derived from dCas12a variants.

a Reciprocal combination of different deaminases, including A3A-Y130F, A3A-N57G, and TadA*8e-V106W, with three dCas12a variants, that is, enCas12a, RR, and RVR, to develop new CBEs and ABEs with expanded PAM recognition. **b** Schematic representation of Cas12a variant-mediated CBEs, with A3A-Y130F or A3A-N57G fused to the N-terminal. **c** Schematic representation of Cas12a variant-mediated ABEs, with TadA*8e-V106W fused to the N-terminal.

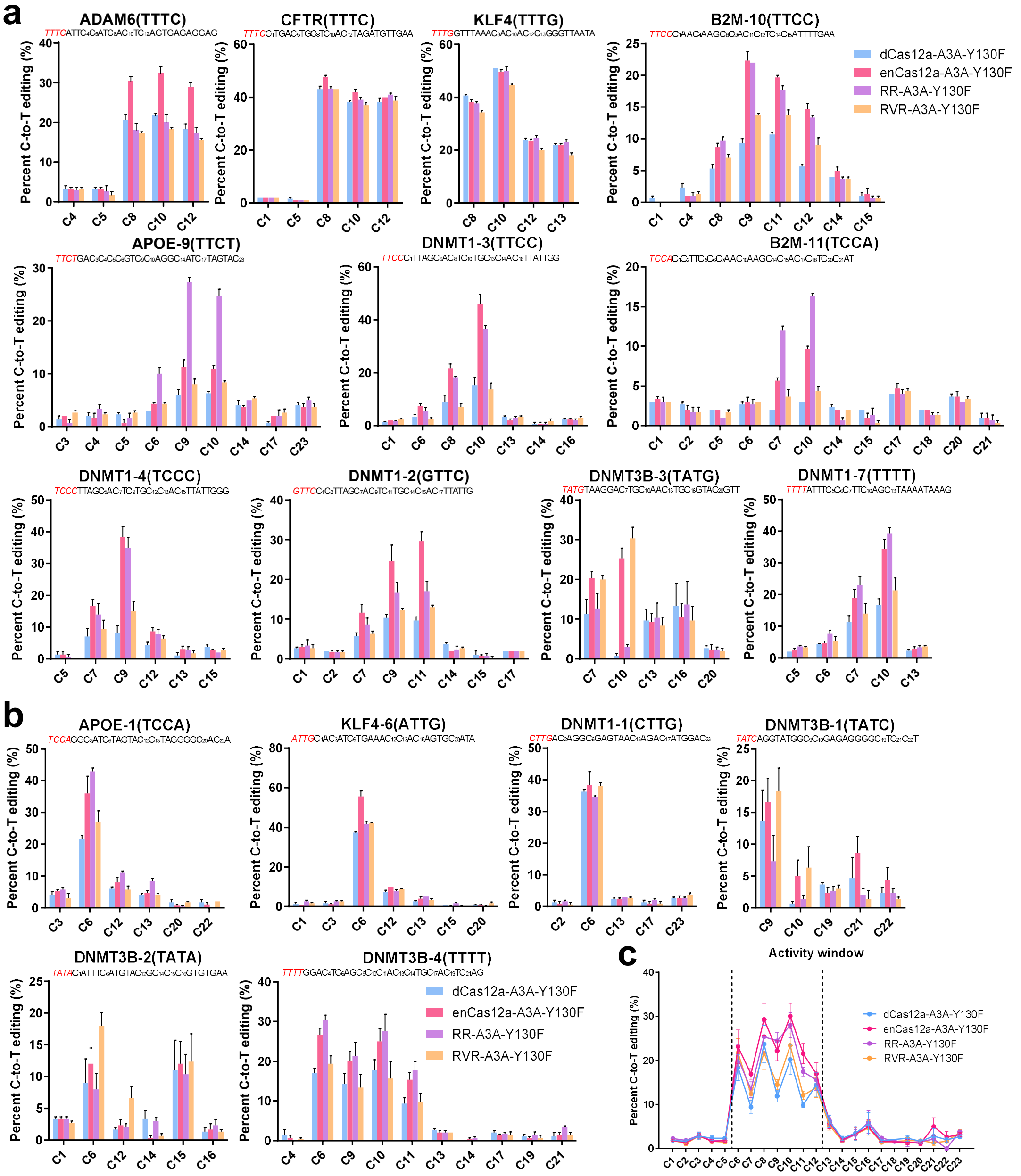

Supplementary Fig. 5 Efficiencies of targeted C-to-T conversion for CBEs with Cas12a variants fused to A3A-Y130F in HEK293T cells.

a, **b** Efficient C-to-T conversion by Cas12a variant-mediated CBEs at endogenous sites with canonical TTTV PAM and alternative PAMs in HEK293T cells (n=3), related to **Figs. 1b–d**. PAM sequences for individual sites are marked in the brackets. **c** Activity windows of different CBEs in HEK293T cells. Values and error bars represent the mean and SEM, respectively.

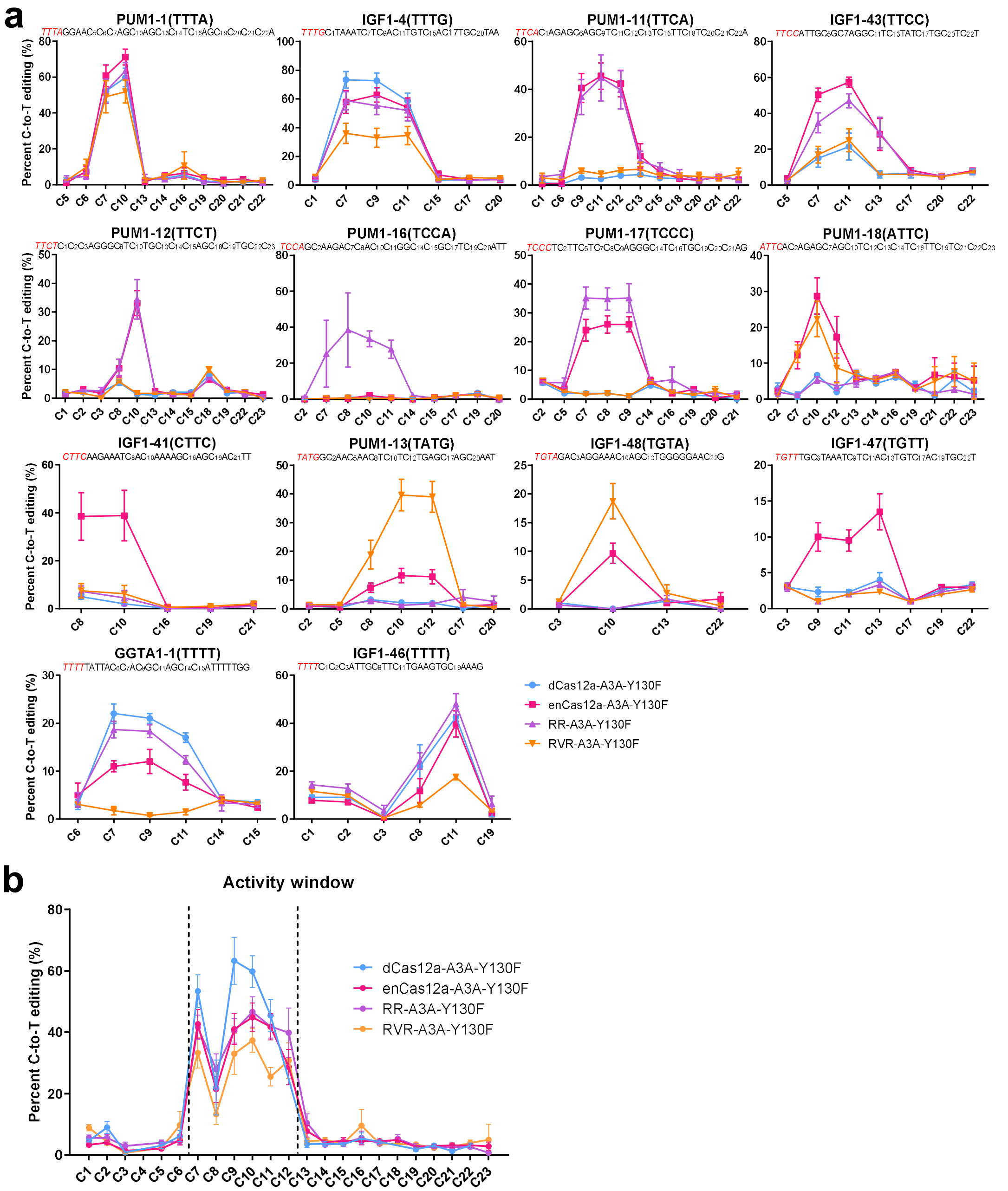

Supplementary Fig. 6 Efficiencies of targeted C-to-T conversion for CBEs with Cas12a variants fused to A3A-Y130F in porcine PA embryos.

a Efficient C-to-T conversion by Cas12a variant-mediated CBEs at endogenous sites with canonical TTTV PAM and alternative PAMs in porcine embryos (n≥2), related to **Figs. 1e–g**. PAM sequences for individual sites are marked in the brackets. **b** Activity windows of different Cas12a variant-CBEs in embryos. Values and error bars represent the mean and SEM, respectively.

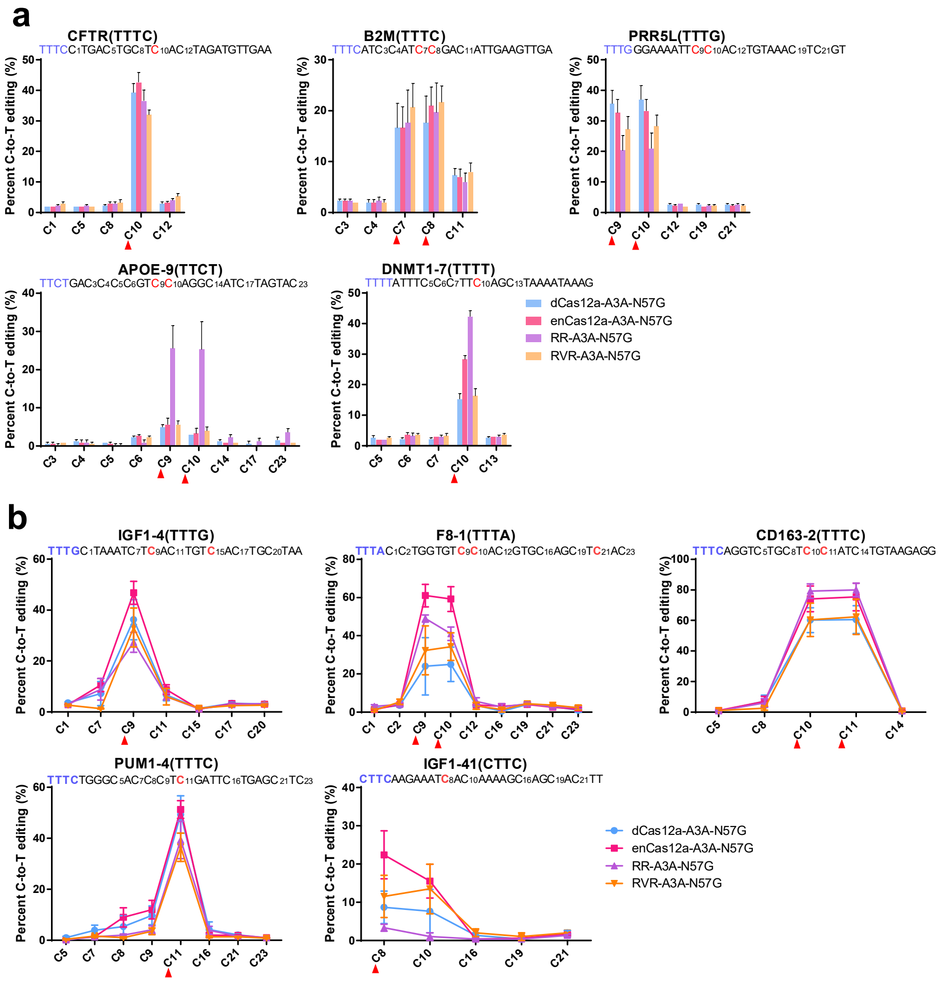

Supplementary Fig. 7 Efficiencies of targeted C-to-T conversion for CBEs with Cas12a variants fused to A3A-N57G.

The A3A-N57G in Cas12a variant-mediated CBEs retained motif preferences for TC/TCCR indicated by red arrowheads, tested both in HEK293T cells (n=3) (**a**) and porcine embryos (n≥2) (**b**). PAM sequences for individual sites are marked in the brackets. Values and error bars represent the mean and SEM, respectively.

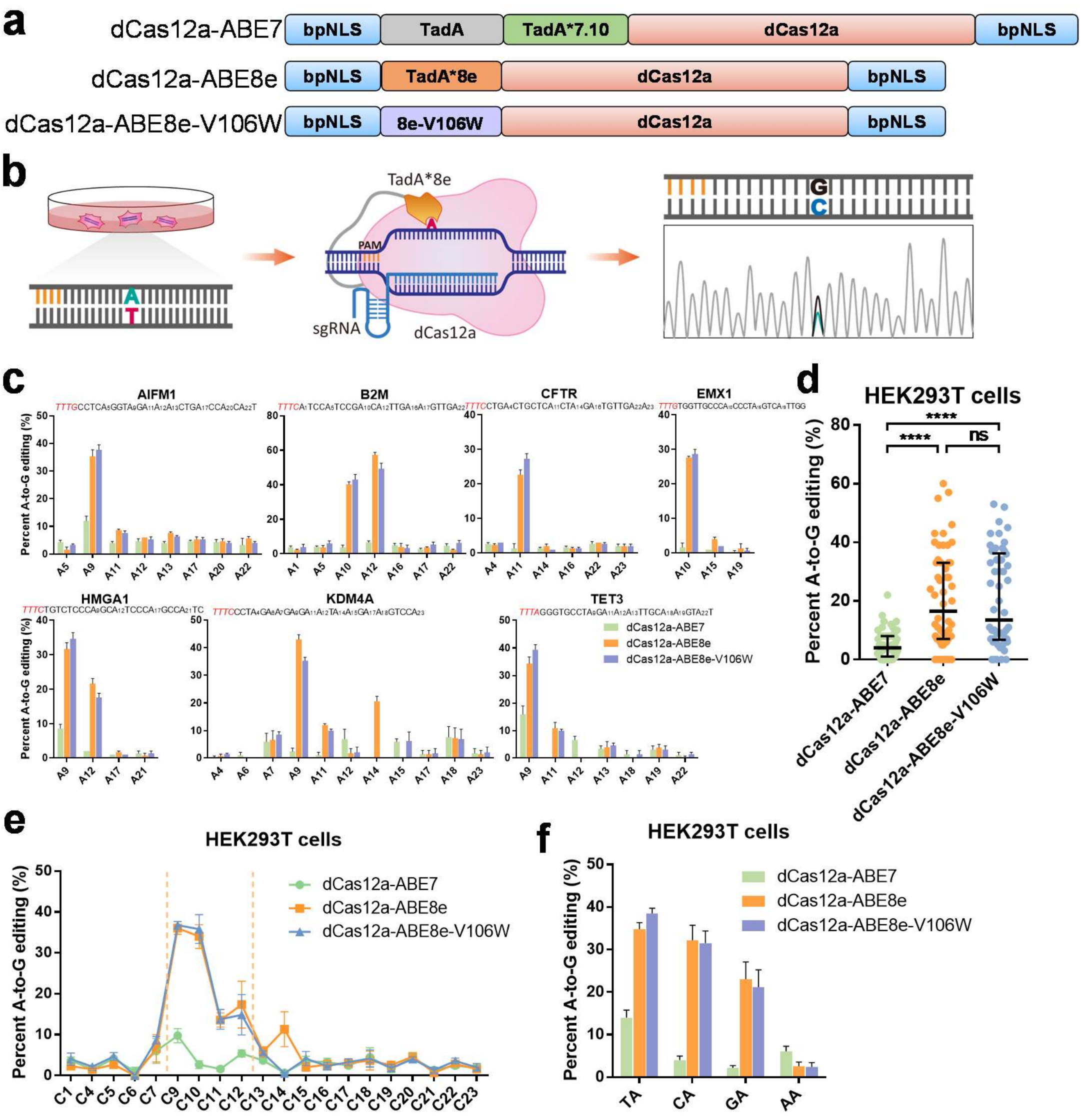

Supplementary Fig. 8 Efficient A-to-G conversions mediated by dCas12a-ABE8e and dCas12a-ABE8e-V106W in HEK293T cells.

a Schematic representation of Cas12a-ABEs with different adenine deaminases fused to the N-terminal of dCas12a. **b** Schematic illustrating A-to-G conversion by base editor dCas12a-ABE8e in cultured cells. **c** A-to-G editing mediated by different Cas12a-ABEs at 7 sites in HEK293T cells (n=3). **d** Average efficiencies of Cas12a-ABEs at 7 sites shown in **c**. **e** Activity windows of different Cas12a-ABEs, with the positions lacking adenine in all 7 tested sites omitted. **f** Sequence preferences of different Cas12a-ABEs. Values and error bars for **c**, **e**, and **f** represent the mean and SEM, respectively. Values for **d** represent the median with interquartile range. Statistical significance was calculated by Kruskal–Wallis test, and Dunn's multiple comparisons test was performed. ns indicates not statistical significant and ****p < 0.0001.

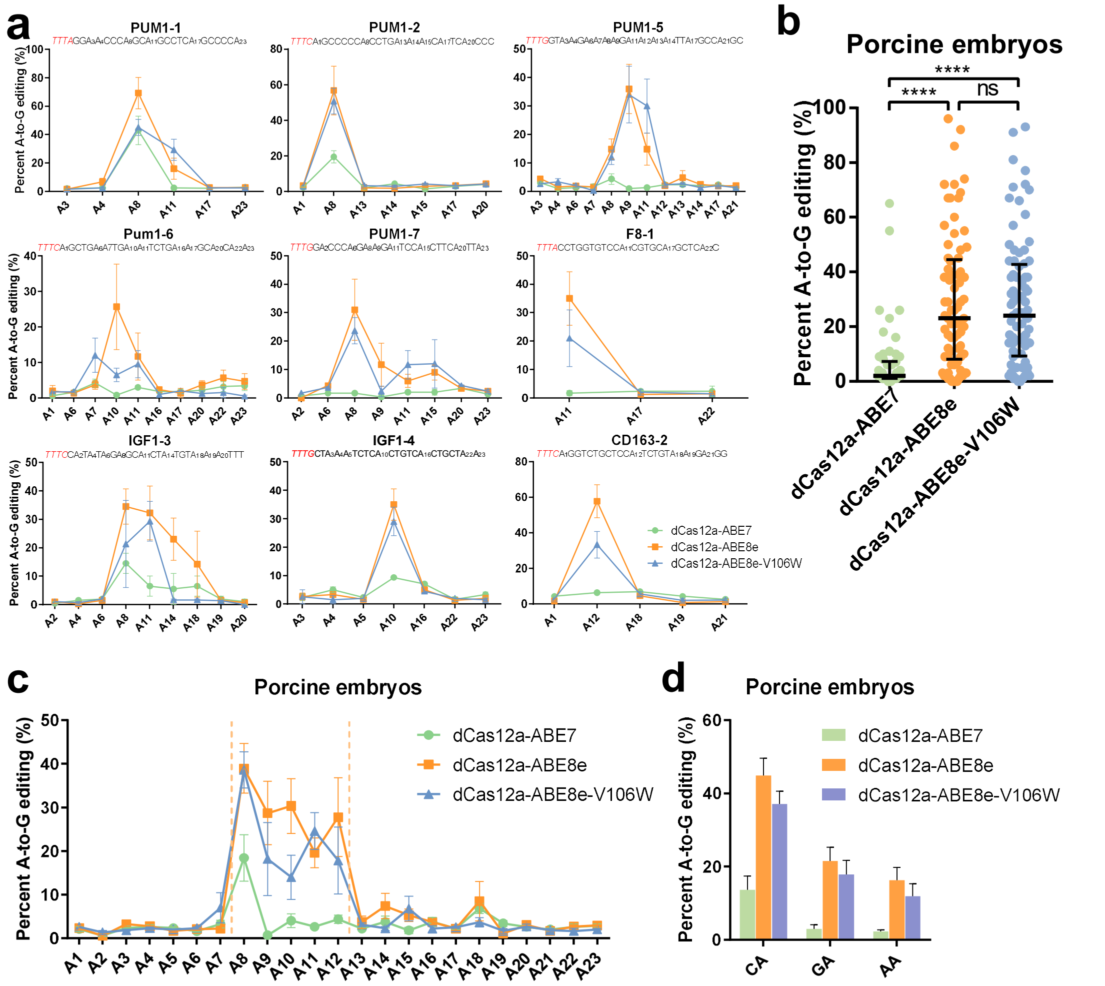

Supplementary Fig. 9 Efficient A-to-G conversions mediated by dCas12a-ABE8e and dCas12a-ABE8e-V106W in porcine PA embryos.
a A-to-G editing mediated by different Cas12a-ABEs at 9 sites in porcine embryos (n≥2). **b** Average efficiencies of Cas12a-ABEs at 9 sites shown in **a**. **c** Activity windows of different Cas12a-ABEs, with the positions lacking adenine in all 9 tested sites omitted. **d** Sequence preferences of different Cas12a-ABEs, noting that TA motif was absent in all 9 tested sites. Values and error bars for **a**, **c**, and **d** represent the mean and SEM, respectively. Values for **b** represent the median with interquartile range. Statistical significance was calculated by Kruskal-Wallis test, and Dunn's multiple comparisons test was performed. ns indicates not statistical significant and ****p < 0.0001.

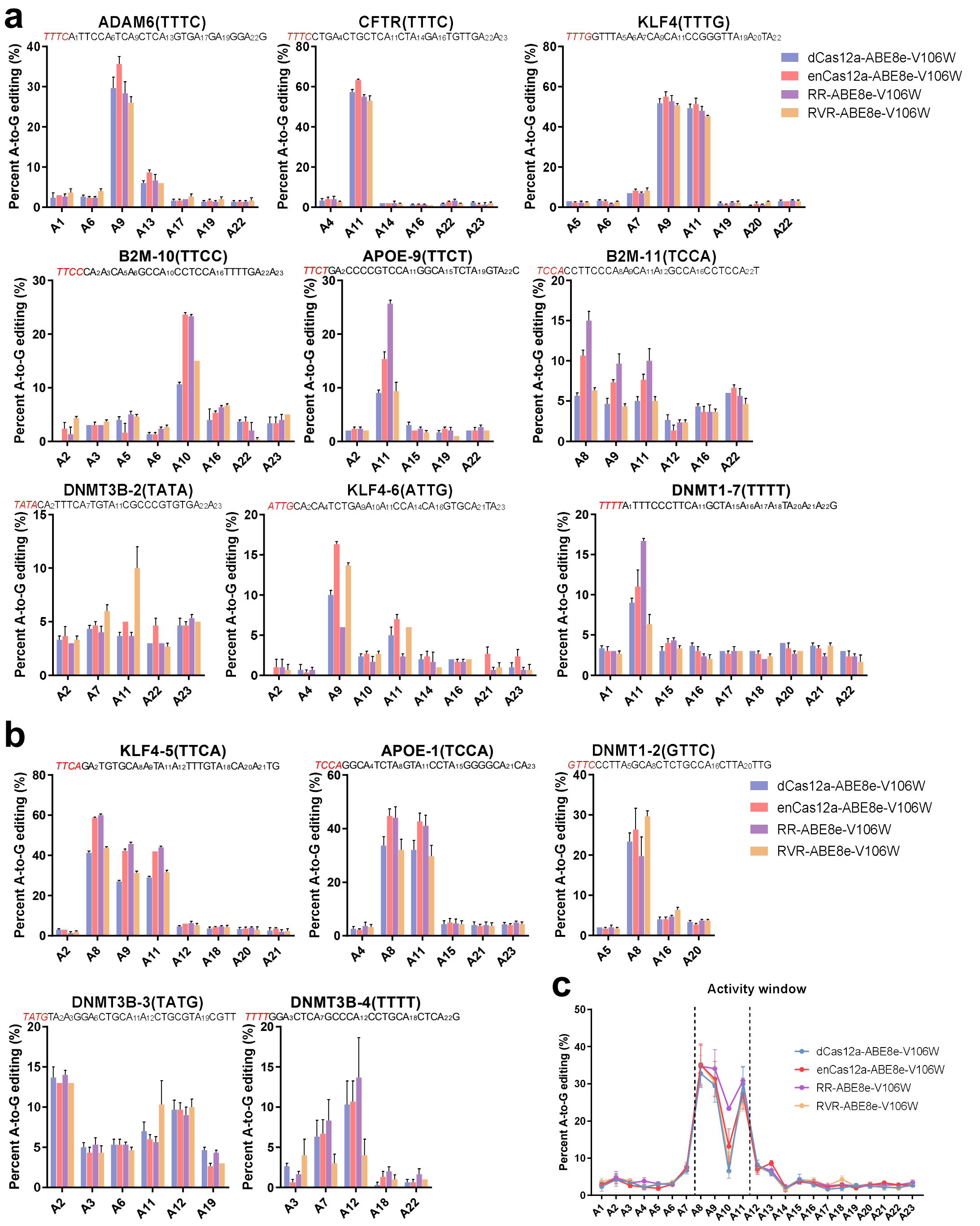

Supplementary Fig. 10 Efficiencies of targeted A-to-G conversion for ABEs with Cas12a variants fused to TadA*8e-V106W in HEK293T cells.

a, **b** Efficient A-to-G conversion by Cas12a variant-mediated ABEs at endogenous sites with canonical TTTV PAM and alternative PAMs in HEK293T cells (n=3), related to **Fig. 2**. PAM sequences for individual sites are marked in the brackets. **c** Activity windows of different ABEs. Values and error bars represent the mean and SEM, respectively.

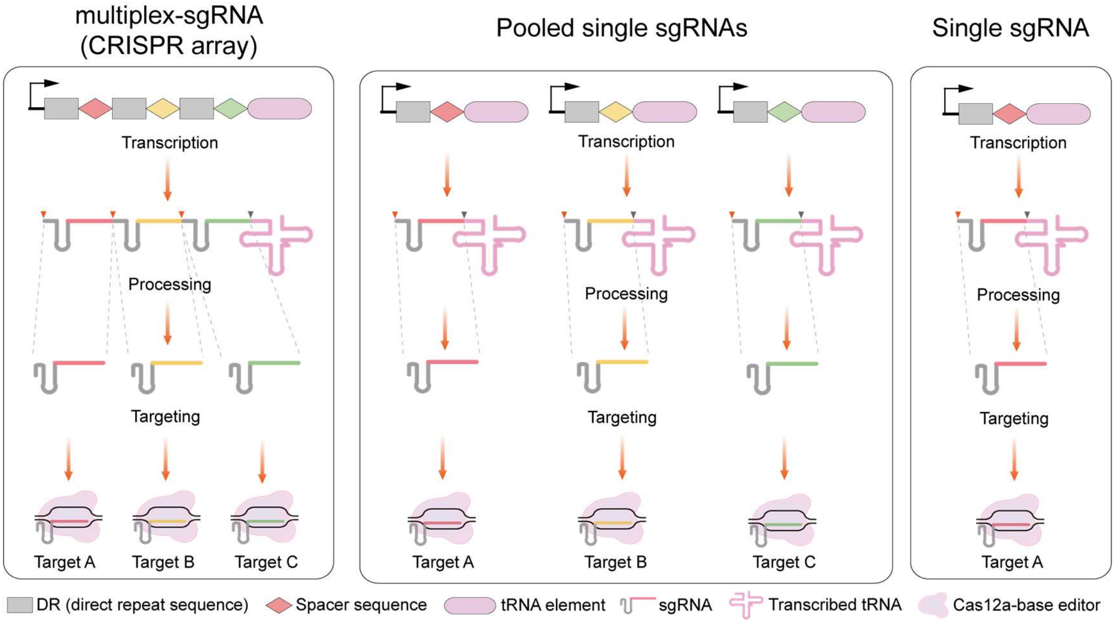

Supplementary Fig. 11 Schematic of multiplexed base editing with individual sgRNAs.

Schematic illustrating multiplexed base editing with multiplex-sgRNA (CRISPR array) or pooled single sgRNAs. Left panel: a single transcript of the tandem sgRNAs from a single plasmid was processed by Cas12a into multiple mature sgRNAs, followed by simultaneous base editing of multiple targets. Middle panel: multiple sgRNA expression plasmids, which transcribed sgRNAs separately, were pooled together for simultaneous base editing of multiple targets. Right panel: a single sgRNA for the corresponding target was used as a control. Orange and gray triangles denote the cleavage sites of Cas12a and RNase P, respectively. A tRNA precursor sequence is appended to downstream of the pre-sgRNA to improve the CRISPR/Cas12a system.

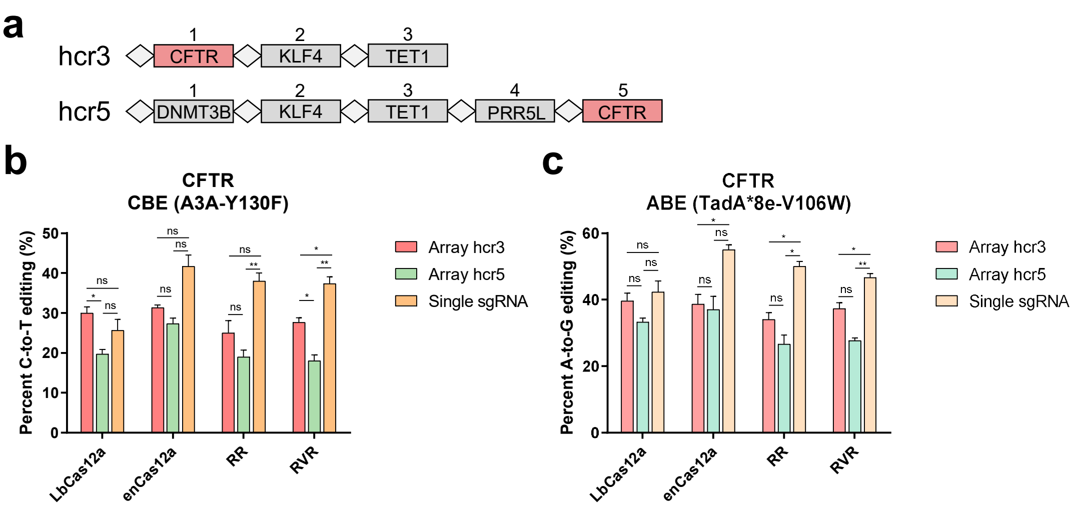

Supplementary Fig. 12 Base editing for target *CFTR* with CRISPR array hcr3 or hcr5

a Constructs of CRISPR arrays hcr3 and hcr5, where the sgRNA targeting *CFTR* is located at the first and fifth (also the last) positions in the arrays, respectively. **b**, **c** Comparison of editing efficiencies at the *CFTR* site with different tandem CRISPR array (hcr3 or hcr5). Cas12a variant-mediated CBEs (**b**) and ABEs (**c**) were determined, with a single sgRNA for *CFTR* as the control (n=3), related to **Figs. 3c–f**. Values and error bars represent the mean and SEM, respectively. Statistical significance was calculated by Welch's ANOVA test, and Tamhane's T2 multiple comparisons test was performed. ns (not significant), $p \geq 0.05$; $^*p < 0.05$; $^{**}p < 0.01$.

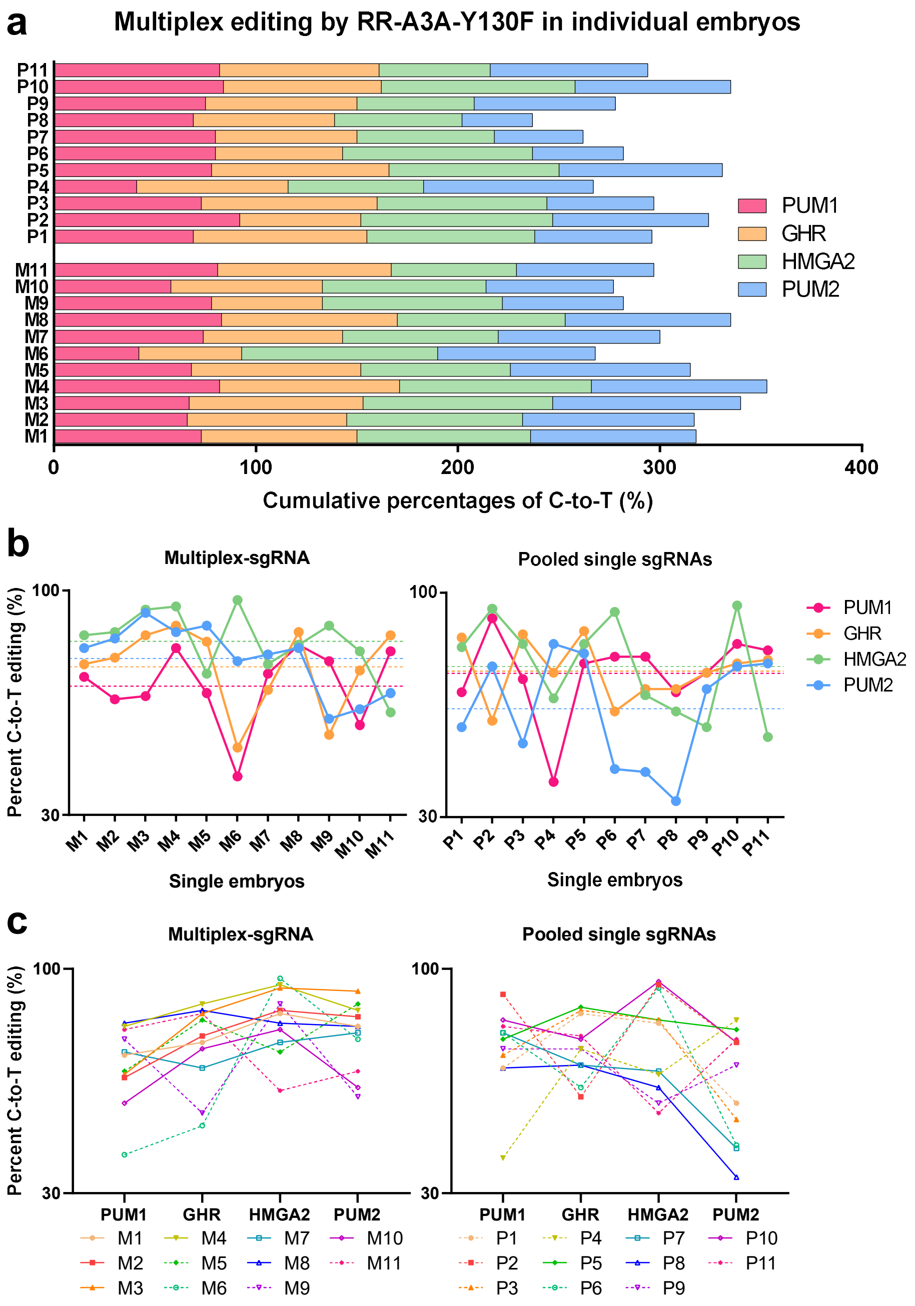

Supplementary Fig. 13 Multiplxed base editing in porcine embryos by RR-A3A-Y130F with multiplex-sgRNA or pooled single sgRNAs.

a Cumulative percentages of C-to-T editing at the four loci (*PUM1*, *GHR*, *HMGA2*, and *PUM2*) with multiplex-sgRNA (CRISPR array pcr4) or pooled four sgRNAs. **b** Efficiencies of C-to-T editing for the four loci among individual single embryos. The dashed lines in different colors indicate the average efficiencies of the corresponding sites. **c** Efficiencies of C-to-T editing for single embryos among the four loci. The dashed and solid lines indicate high and low efficiency variability among the four loci for single embryos, respectively. Related to **Fig. 4**. M1–M11 indicate multiplx-sgRNA, and P1–11 indicate pooled four sgRNAs.

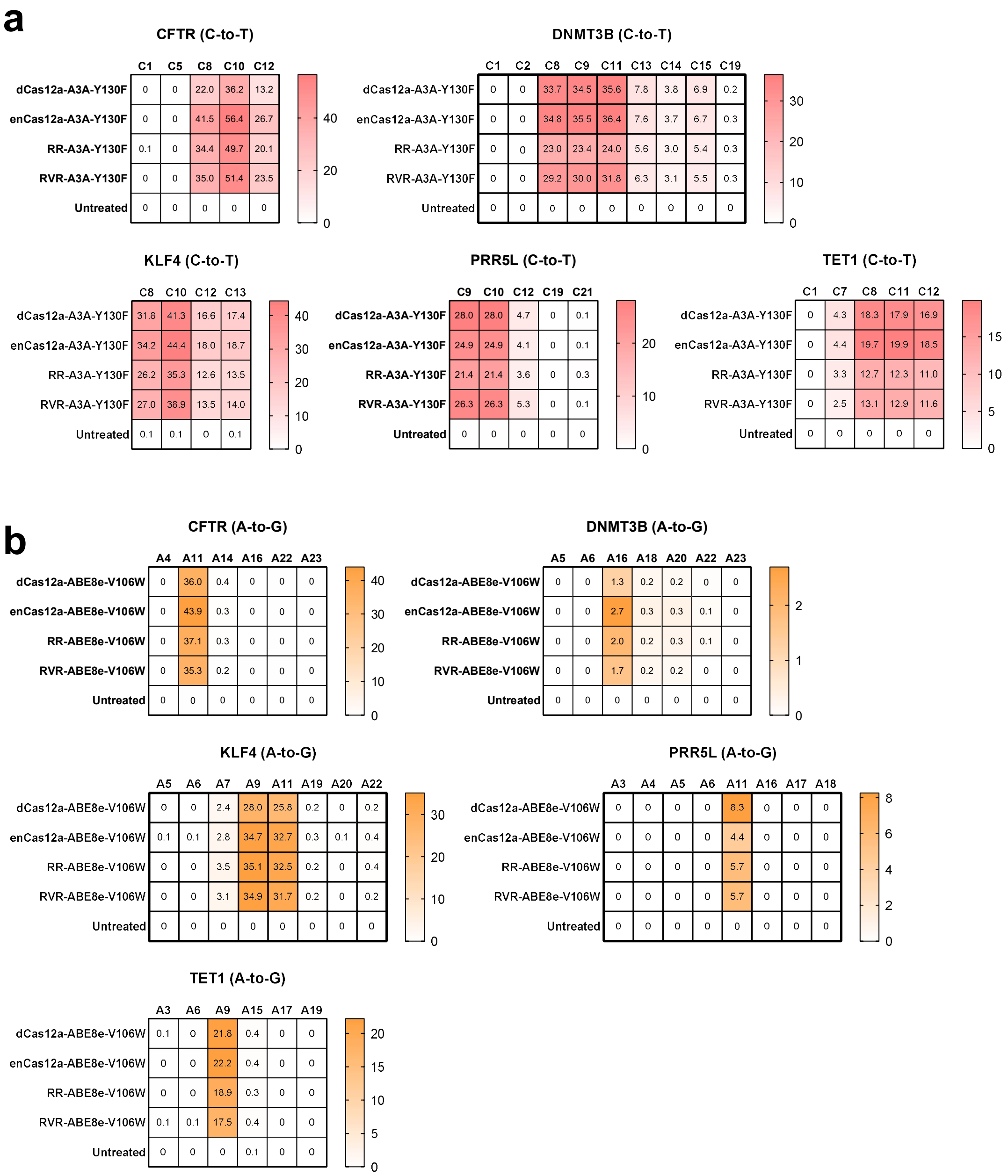

Supplementary Fig. 14 Efficiencies of targeted base editing by Cas12a variant-mediated base editors at the five selected sites in HEK293T cells. **a**, **b** Heat maps showing the frequencies of C-to-T base editing for indicated CBEs (**a**) and A-to-G base editing for indicated ABEs (**b**) at the five selected sites with TTTV PAM, related to **Fig. 5**. Values represent the mean of three independent biological replicates (n=3).

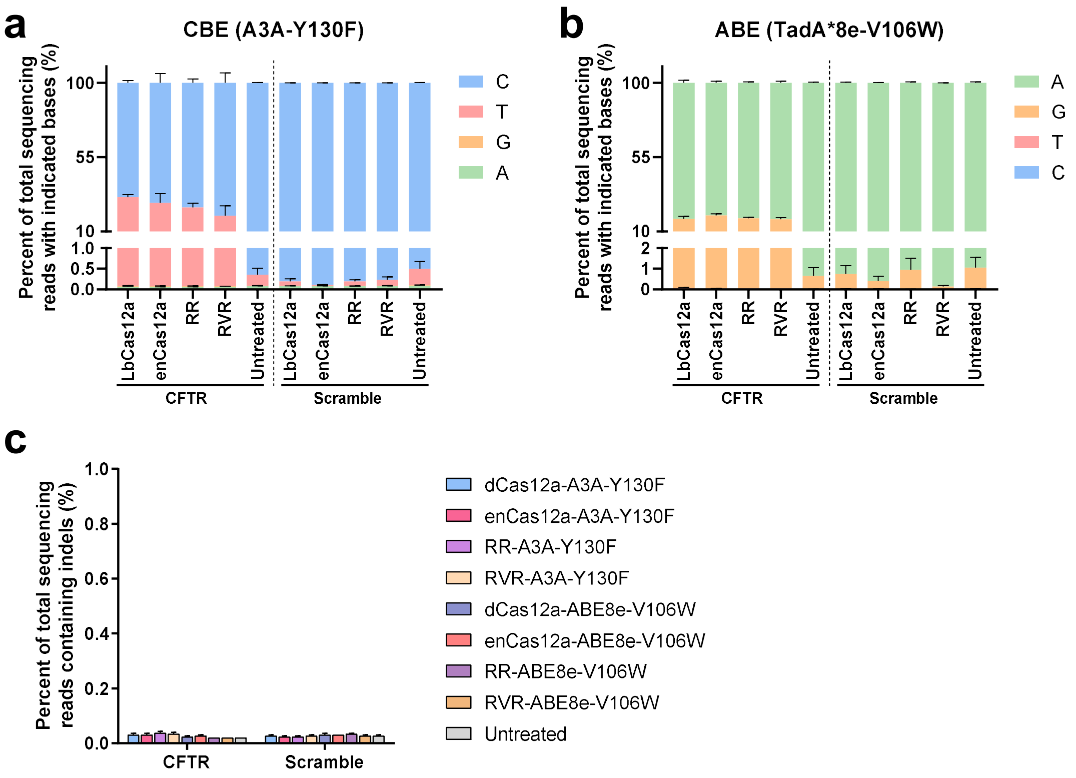

Supplementary Fig. 15 Output comparison of the target sgRNA and scramble sgRNA at the *CFTR* site.

a Frequencies of indicated bases at the positions 10 of *CFTR* for individual CBEs with the target sgRNA or sramble sgRNA. **b** Frequencies of indicated bases at the positions 11 of *CFTR* for individual ABEs with the target sgRNA or sramble sgRNA. **c** Indels frequencies for indicated CBEs and ABEs at the *CFTR* sites with the target sgRNA or scramble sgRNA. The non-targeting scramble sgRNA, which contains the same nucleotide composition as target sgRNA of *CFTR* while does not target the human genome, was used as a negative control. Values represent the mean of three independent biological replicates (n=3). Values and error bars represent the mean and SEM, respectively.

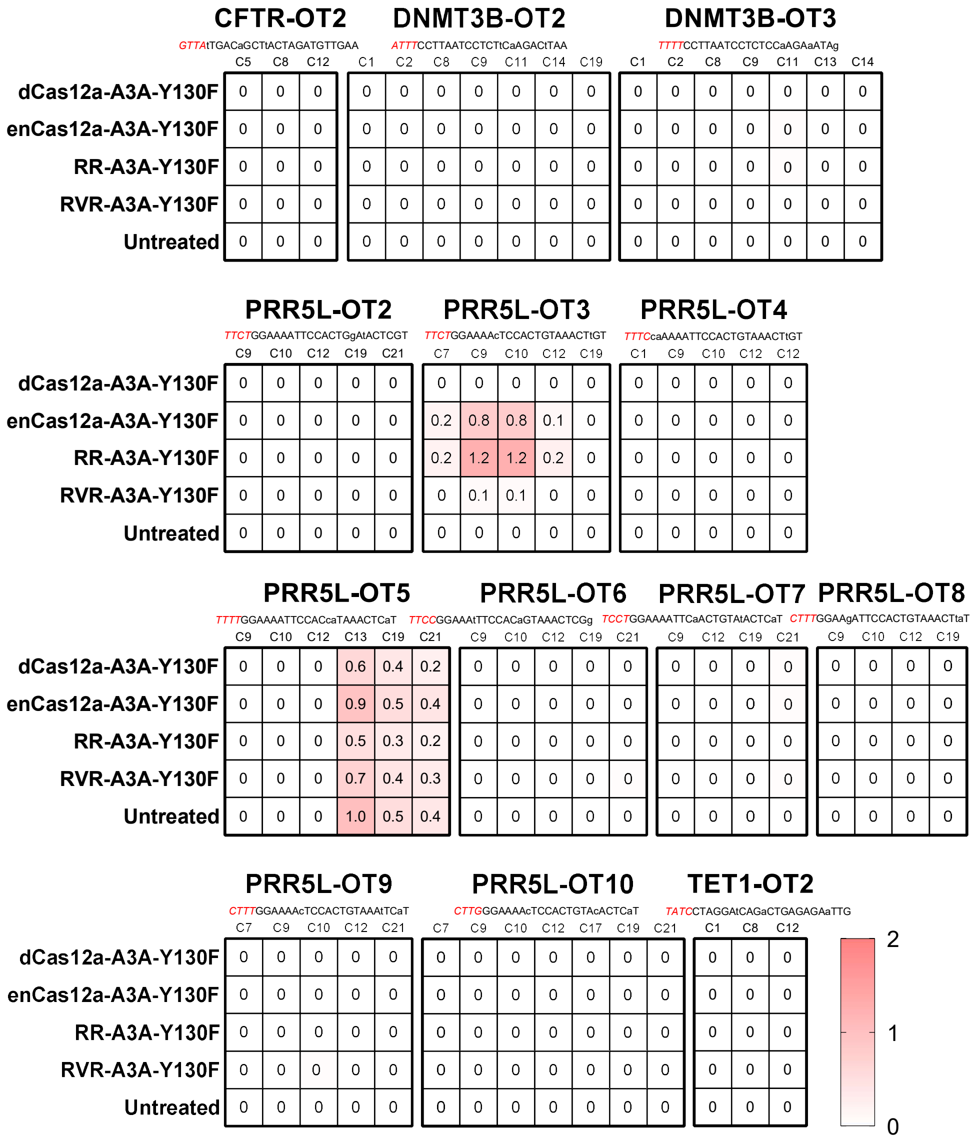

Supplementary Fig. 16 Off-target activity of Cas12a variant-mediated CBEs.

Heat maps showing the Cas12a-dependent C-to-T off-target activity of indicated CBEs at the predicted off-target sites, related to **Fig. 5d**. Values represent the mean of three independent biological replicates (n=3).

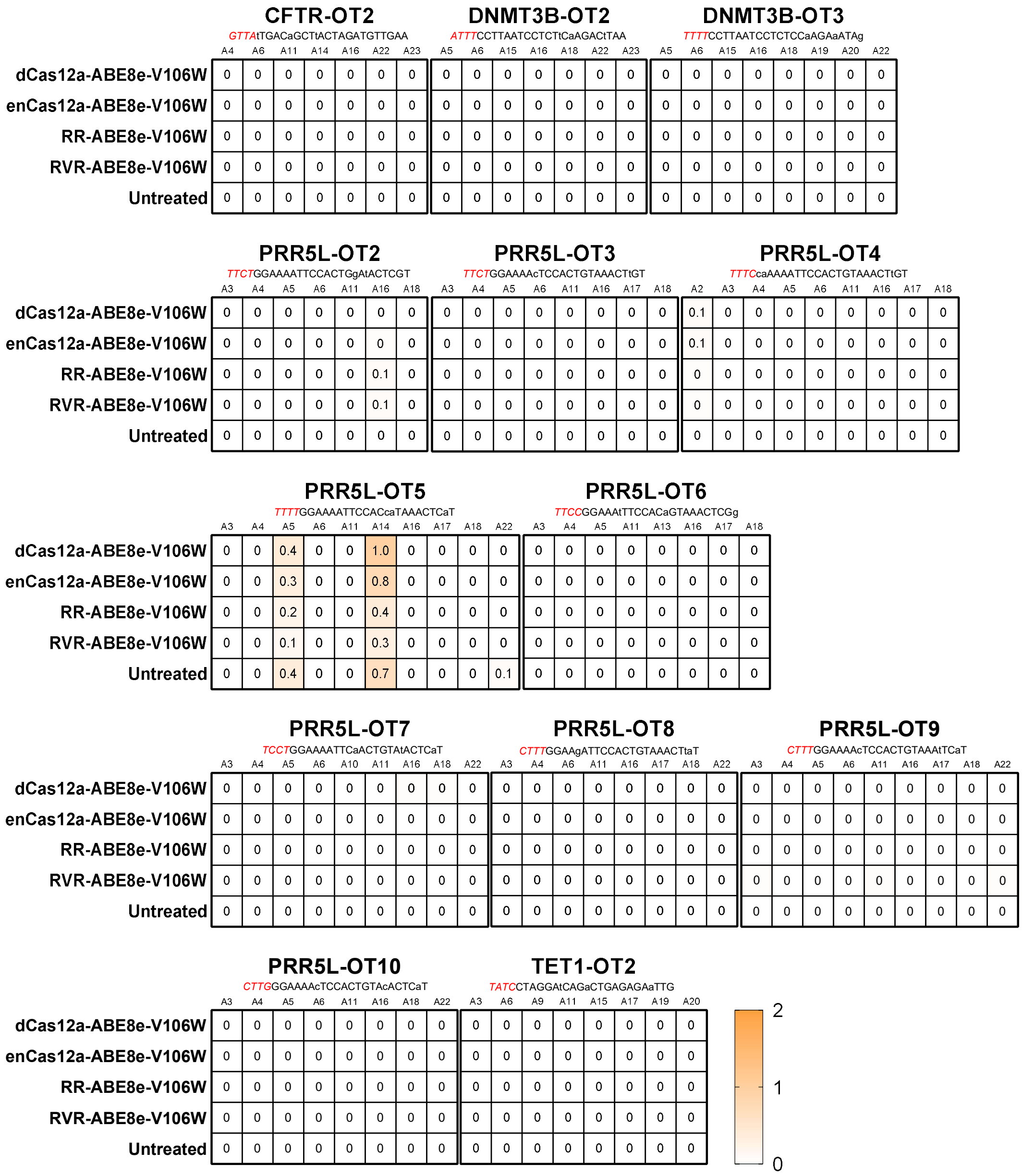

Supplementary Fig. 17 Off-target activity of Cas12a variant-mediated ABEs.

Heat maps showing the Cas12a-dependent A-to-G off-target activity of indicated ABEs at the predicted off-target sites, related to **Fig. 5e**. Values represent the mean of three independent biological replicates (n=3).

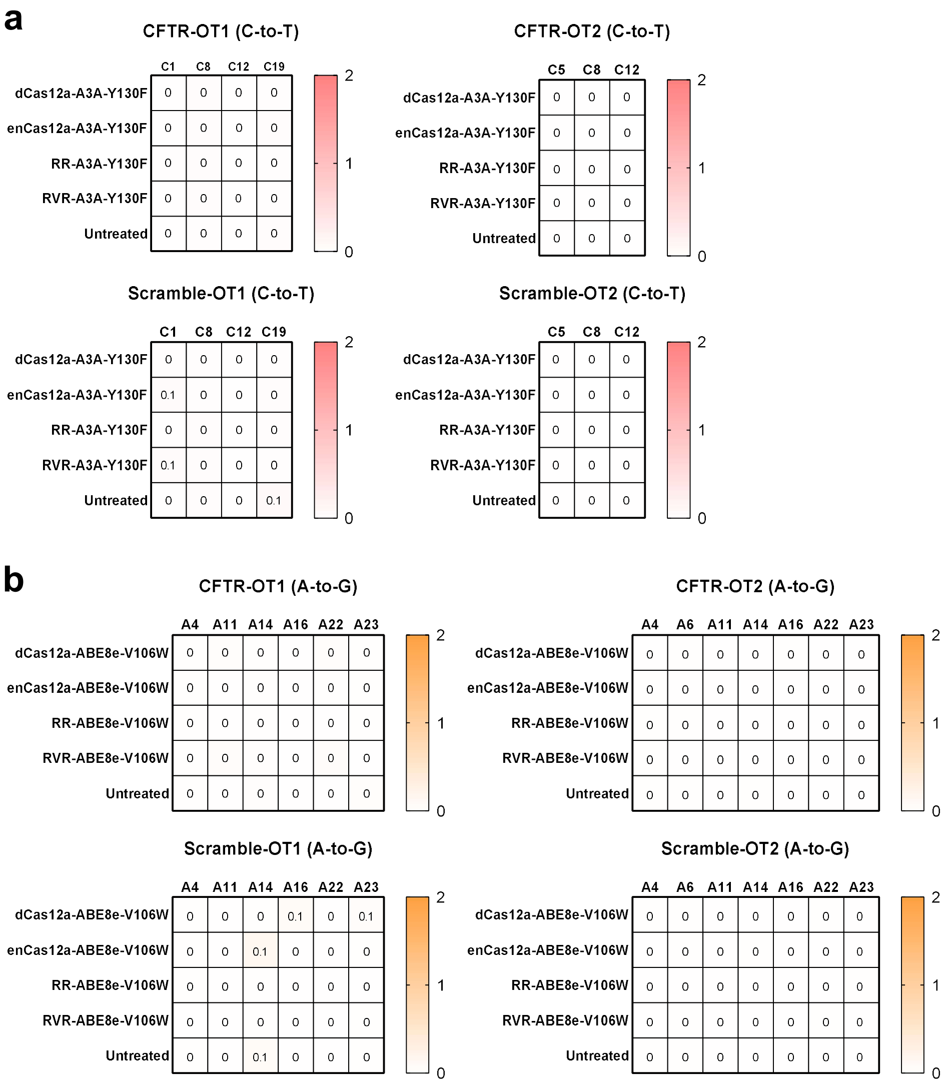

Supplementary Fig. 18 Off-target activity of Cas12a variant-mediated base editors guided by the target sgRNA or scramble sgRNA at the *CFTR* off-target sites.

a, **b** Heat maps showing the Cas12a-dependent C-to-T off-target activity of indicated CBEs (**a**) and A-to-G off-target activity of indicated ABEs (**b**) with the target sgRNA or scramble sgRNA at the predicted off-target sites. The non-targeting scramble sgRNA, which contains the same nucleotide composition as target sgRNA of *CFTR* while does not target the human genome, was used as a negative control. Values represent the mean of three independent biological replicates (n=3).
