# Supplementary material for: Multiplexed base editing through Cas12a variant-mediated cytosine and adenine base editors
Source: Commun Biol. 2022 Nov 2;5:1163. doi: 10.1038/s42003-022-04152-8 (PMC9630288; doi:10.1038/s42003-022-04152-8)
Supplement: Supplementary file 2 — Description of Additional Supplementary Data [file 42003_2022_4152_MOESM2_ESM.docx]

**Description of Additional Supplementary Files**

**File name:** Supplementary Data 1

**Description:** The source data behind the graphs in the paper.

**File name:** Supplementary Data 2

**Description:** Lists of the target sequences and PCR primers used to amplify genomic DNA.

**File name:** Supplementary Data 3

**Description:** Lists of interested on-target sites, corresponding potential off-target sequences, and PCR primers used to amplify genomic DNA.
